# Unveiling the Hidden Challenges: A Systematic Review of Self-Identified Caregiver Support Needs for Older Adults in Canada

**DOI:** 10.3389/phrs.2026.1609117

**Published:** 2026-02-26

**Authors:** Sheila A. Boamah, Hoda Herati, Farzana Akter, Farinaz Havaei, Marie-Lee Yous, Sharon Kaasalainen

**Affiliations:** 1 McMaster University School of Nursing, Hamilton, ON, Canada; 2 The University of British Columbia, Vancouver, BC, Canada

**Keywords:** Canada, informal care, older adults, self-identified caregivers, support needs

## Abstract

**Objective:**

In Canada, over 7.8 million individuals provide care, with nearly one-quarter aged 65 or older. As essential partners in aging, caregivers bridge formal care systems and the broader care economy. With caregiving demands expected to double over the next 30 years, identifying and addressing caregivers’ evolving support needs is critical to sustaining compassionate, connected care. This systematic review aims to document caregivers’ self-identified support needs in delivering quality care.

**Methods:**

A systematic search of bibliographic databases and grey literature was conducted in line with PRISMA guidelines and supplemented by reference mining. Eligible studies were peer-reviewed, published in English between 2020 and 2025; reviews and grey literature were excluded. Selection was managed using Covidence, and methodological quality was assessed independent by two reviewers utilizing Joanna Briggs Institute tools.

**Results:**

Of 3,629 records, 83 studies were included: 59 qualitative, 17 quantitative, and 7 mixed-methods. Five key themes with twelve sub-themes emerged, reflecting caregivers’ needs related to system navigation, inclusive technologies, coordinated care system, emotional and practical, and financial/workplace resources.

**Conclusion:**

Caregivers’ insights highlight priority areas to inform caregiver-centred policies, services, and research that enhance caregiver wellbeing and care quality for older adults.

## Introduction

In line with patterns seen in other industrialized nations, Canada is experiencing a marked demographic shift towards an aging population. The proportion of Canadians aged 65 and older is projected to rise from 18.9% in 2023 to 22.7% by 2040, a trend with significant implications for health and social policy [1]. As life expectancy increases, coupled with advances in medicine and technology, the risk of chronic illness rises, with over one-third of Canadian seniors living with multiple chronic conditions [2]. Many older adults with chronic medical conditions and mobility limitations require varying degrees of support with both basic activities of daily living, like bathing, dressing, and eating, and more complex instrumental tasks such as managing finances, shopping, and transportation [3].

Meeting these growing care needs increasingly depends on unpaid caregivers—including family members, spouse or partners, friends, and neighbours—who provide essential support to persons living with chronic illness, disabilities, or age-related challenges. Referred to hereafter as caregivers, these individuals perform a wide range of responsibilities, from managing daily tasks to offering emotional and physical support [4]. In 2018, approximately 7.8 million Canadians aged 15 and older, representing about 25% of the Canadian population, identified as caregivers. Notably, nearly one-quarter of these individuals, or roughly 1.5 million, were aged 65 or older [5]. Women disproportionately assumed these caregiving responsibilities, highlighting a persistent gender gap in unpaid care work [6]. Despite their indispensable role within the healthcare continuum and their substantial contributions to both societal wellbeing and the care economy, caregivers remain underrecognized and undervalued [7]. Many lack formal training, adequate resources, and systematic assessments of their support needs, resulting in significant physical, emotional, and financial strain as they navigate the complex demands of caregiving [8].

This strain is often compounded by unmet needs—the gap between the support services caregivers consider essential for managing both the care recipient’s condition and their own wellbeing, and the resources they actually have access to [9]. Research indicates that the unmet needs of caregivers adversely affect their mental health and wellbeing, leading to heightened risks of social isolation, loneliness, depression, and anxiety [10, 11], while also reducing caregivers’ capacity to provide high-quality care [12]. In Canada, caregiver needs are addressed at both federal and provincial levels through a range of policy mechanisms, including legislation (e.g., Caregiver Recognition Acts), employment protections (e.g., caregiver leave), financial supports (e.g., tax credits and Employment Insurance caregiving benefits), and health and home care services [13]. Despite these efforts, existing initiatives remain insufficient, as caregiving programs and services are often fragmented, poorly coordinated, and challenging to navigate [14]. This persistent gap between caregiver needs and available supports highlights a critical oversight in care planning and policy development. To design effective, evidence-based support systems, it is imperative to systematically assess and document caregivers’ self-identified needs. This study therefore seeks to address this gap by conducting a systematic review to explore the following research question: What support needs do caregivers of older adults in Canada identify as essential for delivering quality care?

## Methods

We conducted a systematic review of the literature following the Preferred Reporting Items for Systematic reviews and Meta-Analyses (PRISMA) 2020 guidelines, which include a 27-item checklist to promote transparent, complete, and rigorous reporting [15]. A systematic review was selected for its rigorous, transparent, and replicable methodology, which minimizes bias and enhances the reliability of findings—critical for informing evidence-based policy and practice. The Population, Exposure, Outcome (PEO) framework guided the development of our research question, inclusion criteria, and search strategy, supporting a systematic and focused study selection process. The included studies align with the following PEO components: (P) unpaid family or friend caregivers of older adults in Canada, (E) the experience of providing care to individuals with chronic conditions, and (O) the support needs identified by caregivers (e.g., emotional, financial, informational, respite, and practical support).

### Information Sources and Search Strategy

The search strategy for this review was created and modified with the help of a health science librarian. We searched five social and health science databases, including Embase, Scopus, MEDLINE, CINAHL, and PsycINFO, to identify the support needs of caregivers for older adults in Canada until July 2024 (updated in June 2025). In addition, reference lists of eligible articles were hand searched to identify related studies. The full search strategies for all databases are reported as Supplementary Table S1.

### Eligibility Criteria

The search was restricted to original, peer-reviewed research articles published in English between January 2020 and July 2024, employing qualitative, quantitative, or mixed methods designs. This timeframe was selected to manage the volume of literature while maintaining relevance to current practice, and to capture the evolving support needs of caregivers during and after the COVID-19 pandemic, a period marked by significant disruptions to caregiving roles, healthcare access, and support systems in Canada. Eligible studies focused on the support needs of caregivers of older adults. While ≥60 years is a commonly accepted threshold for defining older adults in the Canadian context, studies were also included when caregivers supported older individuals with chronic conditions, even if age was not explicitly reported, to avoid excluding findings relevant to the target population. Studies that did not explicitly focus on older adults, the Canadian context, or were review articles were excluded (see Supplementary Table S2).

### Selection Process

**FIGURE 1:**
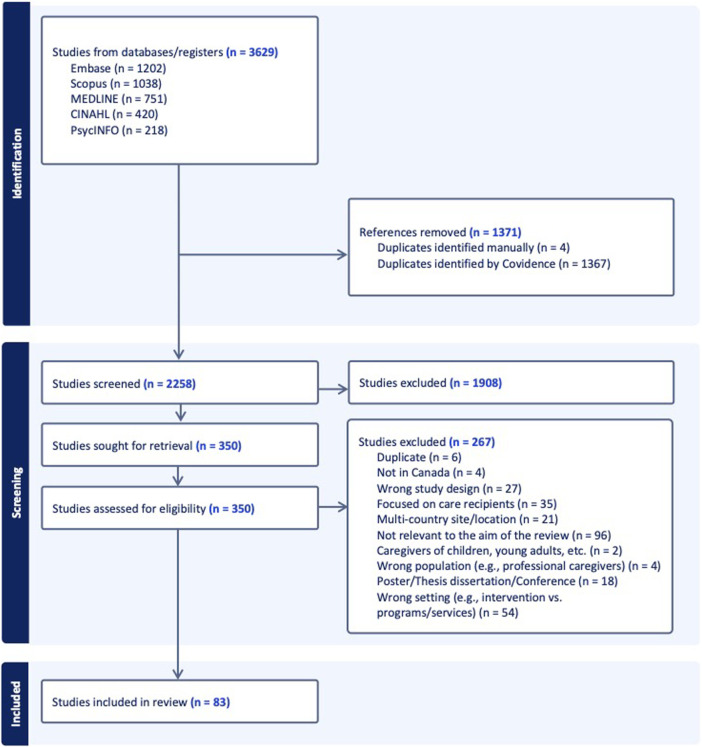
PRISMA flowchart diagram of the selection process (Canada, 2025).

The screening and selection of the article records, data extraction, and quality assessment of the studies (risk of bias assessment) were managed using Covidence web-based software, the recommended platform for the Cochrane Library [16]. Our search identified a total of 3629 articles, 1371 of which were duplicates. Three reviewers (HH, FA, and MA) independently screened the titles and abstracts of 2258 studies based on predetermined inclusion and exclusion criteria. As a result, 1908 studies were excluded, and 350 studies were retrieved for full-text screening, which was conducted independently by the three researchers. Discrepancies were resolved through discussion, when unresolved cases at the full-text stage adjudicated by the lead researcher (SB). In total, 83 studies were included in this review. Figure 1 illustrates the PRISMA flowchart, which shows the selection process of the studies.

### Data Collection/Extraction Process

Data extraction was carried out independently by at least two reviewers using Covidence, a widely utilized platform for screening and data extraction in literature reviews. A modified version of Covidence’s standard “Data extraction template” was adapted by the research team to align with the specific aim of this review. Data from each of the 83 included articles were independently charted by two reviewers and subsequently reviewed by a third reviewer to reach consensus.

### Quality Appraisal (Assessment of Risk of Bias)

The methodological quality of the included qualitative and quantitative studies was assessed by two reviewers (HH, FA) independently using the Joanna Briggs Institute (JBI) critical appraisal tools relevant to each study design [17, 18]. For mixed-method studies, both qualitative and quantitative JBI tools were applied. Given the heterogeneity of study designs, statistical synthesis (e.g., meta-analysis) was not feasible. Hence, quality appraisal was used to systematically summarize each study’s methodological rigour using JBI’s established criteria.

Each tool consists of items addressing key methodological domains (e.g., congruity between research methodology and research questions, adequacy of data collection, and clarity of inclusion criteria). For scoring purposes, each item was rated as “Yes” (criterion met), “No” (criterion not met), “Unclear,” or “Not Applicable”. While JBI does not prescribe fixed cut-off scores for categorizing methodological quality, many systematic reviews have used adapted scoring systems with researcher-defined thresholds to classify studies as low, moderate, or high quality [19–21]. In our systematic review, the quality appraisal scoring system was researcher-defined: each item rated as “Yes” was assigned 1 point, while responses of “No” or “Unclear” received 0 points. No appraisal items were rated as “Not Applicable.” Ultimately, a total appraisal score was assigned by adding the number of “Yes” responses, with higher total scores indicating greater methodological rigour. No studies were excluded based on quality; however, appraisal scores were used to inform the interpretation of findings in the synthesis, particularly in assessing the strength and trustworthiness of the evidence [22]. Table 1 presents the quality appraisal scoring categories for the different study designs included in our review.

**TABLE 1:** Quality appraisal scoring categories (systematic review, Canada, 2020–2025).

Study design	Quality appraisal scoring categories
Qualitative	1–4: Low; 5–7: Moderate; 8–10: High
Quantitative (cross-sectional)	1–3: Low; 4–6: Moderate; 7–8: High
Mixed-methods	1–6: Low; 7–12: Moderate; 13–18: High

## Results

### Description of Included Studies

A summary of the characteristics of the included studies is presented in Supplementary Table S3. Out of 83 studies, 59 (71%) were qualitative, 17 (21%) were quantitative (cross-sectional), and 7 (8%) were mixed-method studies. The studies were conducted across multiple Canadian provinces:^1^ 33 in Ontario (40%), 10 in Quebec (12%), 10 in Alberta (12%), 6 in British Columbia (7%), 6 in Saskatchewan (7%), 4 in Nova Scotia (4%), 2 in New Brunswick (2%), and one in each of Prince Edward Island, Manitoba, and Yukon Territory. Additionally, 17 studies were national in scope (20%). The retained studies included between 5 and 6,674 participants. Care recipients resided in community settings, LTC facilities, or hospitals and presented with a range of conditions, including neurodegenerative diseases (e.g., Alzheimer’s disease, dementia), life-limiting illnesses (e.g., cardiovascular diseases, stroke, cancer), chronic physical conditions (e.g., mobility-related difficulties, aging-related frailty), and palliative care needs. Neurodegenerative or cognitive impairment were the most commonly reported diagnosis for the care recipients (45%). In line with the review’s focus on system-level interventions and policy implications, data were not stratified by caregiver age, gender, or care setting.

Regarding quality appraisal, of the 59 qualitative studies assessed, two were classified as moderate quality, while all of the remaining were scored as high quality. Among the 17 quantitative (which were all cross-sectional) studies, 11 (65%) were rated as high quality, while the remaining six (35%) were of moderate quality. Of the seven mixed-method studies, six (86%) were rated as high quality and one as moderate quality (14%). A detailed breakdown of quality scores for each JBI appraisal item, as well as the overall quality ratings across study designs, is presented in Tables 2–4.

**TABLE 2:** Quality appraisal of qualitative studies (systematic review, Canada, 2020–2025).

Author(s), year	1. Is there congruity between the stated philosophical perspective and the research methodology?	2. Is there congruity between the research methodology and the research question or objectives?	3. Is there congruity between the research methodology and the methods used to collect data?	4. Is there congruity between the research methodology and the representation and analysis of data?	5. Is there congruity between the research methodology and the interpretation of results?	6. Is there a statement locating the researcher culturally or theoretically?	7. Is the influence of the researcher on the research, and vice-versa, addressed?	8. Are participants, and their voices, adequately represented?	9. Is the research ethical according to current criteria or, for recent studies, and is there evidence of ethical approval by an appropriate body?	10. Do the conclusions drawn in the research report flow from the analysis, or interpretation, of the data?	Total score (out of 10)
Ashbourne et al. [60]	Yes (1)	Yes (1)	Yes (1)	Yes (1)	Yes (1)	Yes (1)	Unclear (0)	Yes (1)	Yes (1)	Yes (1)	9
Barber et al. [33]	Yes (1)	Yes (1)	Yes (1)	Yes (1)	Yes (1)	Unclear (0)	Unclear (0)	Yes (1)	Yes (1)	Yes (1)	8
Bélanger-Dibblee et al. [58]	Yes (1)	Yes (1)	Yes (1)	Yes (1)	Yes (1)	Yes (1)	Yes (1)	Yes (1)	Yes (1)	Yes (1)	10
Boamah et al. [64]	Yes (1)	Yes (1)	Yes (1)	Yes (1)	Yes (1)	Yes (1)	Yes (1)	Yes (1)	Yes (1)	Yes (1)	10
Boamah et al. [75]	Yes (1)	Yes (1)	Yes (1)	Yes (1)	Yes (1)	Unclear (0)	Yes (1)	Yes (1)	Yes (1)	Yes (1)	9
Bourbonnais et al. [57]	Yes (1)	Yes (1)	Yes (1)	Yes (1)	Yes (1)	Unclear (0)	Yes (1)	Yes (1)	Yes (1)	Yes (1)	9
Chu et al. [79]	Yes (1)	Yes (1)	Yes (1)	Yes (1)	Yes (1)	Yes (1)	Yes (1)	Yes (1)	Yes (1)	Yes (1)	10
Conklin et al. [74]	Yes (1)	Yes (1)	Yes (1)	Yes (1)	Yes (1)	Unclear (0)	Unclear (0)	Yes (1)	Yes (1)	Yes (1)	8
Cooper et al. [30]	Yes (1)	Yes (1)	Yes (1)	Yes (1)	Yes (1)	Yes (1)	Unclear (0)	Yes (1)	Yes (1)	Yes (1)	9
Cruz et al. [27]	Yes (1)	Yes (1)	Yes (1)	Yes (1)	Yes (1)	Unclear (0)	No (0)	Yes (1)	Yes (1)	Yes (1)	8
Dale et al. [52]	Yes (1)	Yes (1)	Yes (1)	Yes (1)	Yes (1)	No (0)	No (0)	Yes (1)	Yes (1)	Yes (1)	8
Ding et al. [91]	Yes (1)	Yes (1)	Yes (1)	Yes (1)	Yes (1)	No (0)	No (0)	Yes (1)	Yes (1)	Yes (1)	8
Elliott [46]	Yes (1)	Yes (1)	Yes (1)	Yes (1)	Yes (1)	No (0)	No (0)	Yes (1)	Yes (1)	Yes (1)	8
Elliot et al. [43]	Yes (1)	Yes (1)	Yes (1)	Yes (1)	Yes (1)	Unclear (0)	Unclear (0)	Yes (1)	Yes (1)	Yes (1)	8
Hande et al. [133]	Yes (1)	Yes (1)	Yes (1)	Yes (1)	Yes (1)	No (0)	No (0)	Yes (1)	Yes (1)	Yes (1)	8
Flemons et al. [29]	Yes (1)	Yes (1)	Yes (1)	Yes (1)	Yes (1)	Unclear (0)	Unclear (0)	Yes (1)	Yes (1)	Yes (1)	9
Fox et al. [55]	Yes (1)	Yes (1)	Yes (1)	Yes (1)	Yes (1)	Yes (1)	Unclear (0)	Yes (1)	Yes (1)	Yes (1)	9
Garnett et al. [31]	Yes (1)	Yes (1)	Yes (1)	Yes (1)	Yes (1)	Yes (1)	Unclear (0)	Yes (1)	Yes (1)	Yes (1)	9
Gibson et al. [110]	Yes (1)	Yes (1)	Yes (1)	Yes (1)	Yes (1)	Yes (1)	Yes (1)	Yes (1)	Yes (1)	Yes (1)	10
Guité-Verret et al. [70]	Yes (1)	Yes (1)	Yes (1)	Yes (1)	Yes (1)	Yes (1)	Yes (1)	Yes (1)	Yes (1)	Yes (1)	10
Hall et al. [34]	Yes (1)	Yes (1)	Yes (1)	Yes (1)	Yes (1)	Unclear (0)	No (0)	Yes (1)	Yes (1)	Yes (1)	8
Hall et al. [134]	Yes (1)	Yes (1)	Yes (1)	Yes (1)	Yes (1)	Unclear (0)	Unclear (0)	Yes (1)	Yes (1)	Yes (1)	8
Holland [89]	Yes (1)	Yes (1)	Yes (1)	Yes (1)	Yes (1)	Yes (1)	Yes (1)	Yes (1)	Unclear (0)	Yes (1)	9
Isenberg et al. [48]	Yes (1)	Yes (1)	Yes (1)	Yes (1)	Yes (1)	No (0)	No (0)	Yes (1)	Yes (1)	Yes (1)	8
Istanboulian et al. [52]	Yes (1)	Yes (1)	Yes (1)	Yes (1)	Unclear (0)	Yes (1)	Yes (1)	Yes (1)	Yes (1)	Yes (1)	9
Kokorelias et al. [39]	Yes (1)	Yes (1)	Yes (1)	Yes (1)	Yes (1)	Yes (1)	Yes (1)	Yes (1)	Yes (1)	Yes (1)	10
Kuluski et al. [134]	Yes (1)	Yes (1)	Yes (1)	Yes (1)	Yes (1)	Yes (1)	Yes (1)	Yes (1)	Yes (1)	Yes (1)	10
Law et al. [32]	Yes (1)	Yes (1)	Yes (1)	Yes (1)	Yes (1)	Unclear (0)	Yes (1)	Yes (1)	Yes (1)	Yes (1)	9
Lee [83]	Yes (1)	Yes (1)	Yes (1)	Yes (1)	Yes (1)	Unclear (0)	Yes (1)	Yes (1)	Yes (1)	Yes (1)	9
Leslie et al. [59]	Yes (1)	Yes (1)	Yes (1)	Yes (1)	Yes (1)	Unclear (0)	Unclear (0)	Yes (1)	Yes (1)	Yes (1)	8
Leung et al. [42]	Yes (1)	Yes (1)	Yes (1)	Yes (1)	Yes (1)	Yes (1)	Yes (1)	Yes (1)	Yes (1)	Yes (1)	10
Luymes et al. [54]	Yes (1)	Yes (1)	Yes (1)	Yes (1)	Yes (1)	Unclear (0)	Unclear (0)	Yes (1)	Yes (1)	Yes (1)	8
Marani et al. [90]	Yes (1)	Yes (1)	Yes (1)	Yes (1)	Yes (1)	Unclear (0)	Yes (1)	Yes (1)	Yes (1)	Yes (1)	9
MacLeod et al. [53]	Yes (1)	Yes (1)	Yes (1)	Yes (1)	Yes (1)	Yes (1)	Yes (1)	Yes (1)	Yes (1)	Yes (1)	10
McKenna et al. [37]	Yes (1)	Yes (1)	Yes (1)	Yes (1)	Yes (1)	Yes (1)	Yes (1)	Yes (1)	Yes (1)	Yes (1)	10
Meng et al. [135]	Yes (1)	Yes (1)	Yes (1)	Yes (1)	Yes (1)	No (0)	No (0)	Yes (1)	Yes (1)	Yes (1)	8
Motta-Ochoa et al. [62]	Yes (1)	Yes (1)	Yes (1)	Yes (1)	Yes (1)	Yes (1)	Yes (1)	Yes (1)	Yes (1)	Yes (1)	10
Obegu et al. [136]	Yes (1)	Yes (1)	Yes (1)	Yes (1)	Yes (1)	No (0)	No (0)	Yes (1)	Yes (1)	Yes (1)	8
Obegu et al. [136]	Yes (1)	Yes (1)	Yes (1)	Yes (1)	Yes (1)	No (0)	No (0)	Yes (1)	Yes (1)	Yes (1)	8
Peckham et al. [78]	Yes (1)	Yes (1)	Yes (1)	Yes (1)	Yes (1)	Yes (1)	Yes (1)	Yes (1)	Yes (1)	Yes (1)	10
Roach et al. [41]	Yes (1)	Yes (1)	Yes (1)	Yes (1)	Yes (1)	Yes (1)	Yes (1)	Yes (1)	Yes (1)	Yes (1)	10
Ravensbergen et al. [92]	Yes (1)	Yes (1)	Yes (1)	Yes (1)	Yes (1)	Unclear (0)	No (0)	Yes (1)	Yes (1)	Yes (1)	8
Reid et al. [69]	Yes (1)	Yes (1)	Yes (1)	Yes (1)	Yes (1)	No (0)	No (0)	Yes (1)	Yes (1)	Yes (1)	8
Savoie et al. [34]	Yes (1)	Yes (1)	Yes (1)	Yes (1)	Yes (1)	Unclear (0)	No (0)	Yes (1)	Yes (1)	Yes (1)	8
Saragosa et al. [44]	Yes (1)	Yes (1)	Yes (1)	Yes (1)	Yes (1)	Unclear (0)	Unclear (0)	Yes (1)	Yes (1)	Yes (1)	8
Sethi [65]	Yes (1)	Yes (1)	Yes (1)	Yes (1)	Yes (1)	Yes (1)	Yes (1)	Yes (1)	Yes (1)	Yes (1)	10
Silverman [86]	Yes (1)	Yes (1)	Yes (1)	Yes (1)	Yes (1)	Unclear (0)	Unclear (0)	Yes (1)	Yes (1)	Yes (1)	8
Smolej et al. [81]	Yes (1)	Yes (1)	Yes (1)	Yes (1)	Yes (1)	No (0)	Yes (1)	Yes (1)	Yes (1)	Yes (1)	9
Stajduhar et al. [84]	Yes (1)	Yes (1)	Yes (1)	Yes (1)	Yes (1)	Yes (1)	Unclear (0)	Yes (1)	Yes (1)	Yes (1)	9
Stolee et al. [63]	Yes (1)	Yes (1)	Yes (1)	Yes (1)	Yes (1)	Yes (1)	Yes (1)	Yes (1)	Yes (1)	Yes (1)	10
Sun et al. [56]	Yes (1)	Yes (1)	Yes (1)	Yes (1)	Yes (1)	Yes (1)	Yes (1)	Yes (1)	Yes (1)	Yes (1)	10
Tseung et al. [77]	Yes (1)	Yes (1)	Yes (1)	Yes (1)	Yes (1)	Unclear (0)	Unclear (0)	Yes (1)	Yes (1)	Yes (1)	8
Wang et al. [66]	Yes (1)	Yes (1)	Yes (1)	Yes (1)	Yes (1)	Yes (1)	Yes (1)	Yes (1)	Yes (1)	Yes (1)	10
Webber et al. [138]	Yes (1)	Yes (1)	Yes (1)	Yes (1)	Yes (1)	Unclear (0)	Yes (1)	Yes (1)	Yes (1)	Yes (1)	9
Weeks et al. [68]	Yes (1)	Yes (1)	Yes (1)	Yes (1)	Yes (1)	Unclear (0)	Unclear (0)	Yes (1)	Yes (1)	Yes (1)	8
Williams et al. [50]	Yes (1)	Yes (1)	Yes (1)	Yes (1)	Yes (1)	No (0)	Unclear (0)	Yes (1)	Yes (1)	Yes (1)	8
Wilson et al. [139]	Yes (1)	Yes (1)	Yes (1)	Unclear (0)	Yes (1)	No (0)	No (0)	Yes (1)	Yes (1)	Yes (1)	7
Yagelniski et al. [49]	Yes (1)	Yes (1)	Yes (1)	Yes (1)	Yes (1)	Unclear (0)	No (0)	Yes (1)	Yes (1)	Yes (1)	8
Yakerson [23]	Yes (1)	Yes (1)	Yes (1)	Yes (1)	Yes (1)	No (0)	No (0)	Yes (1)	Unclear (0)	Yes (1)	7

**TABLE 3:** Quality appraisal of quantitative studies (systematic review, Canada, 2020–2025).

Author(s), year	1. Were the criteria for inclusion in the sample clearly defined?	2. Were the study subjects and the setting described in detail?	3. Was the exposure measured in a valid and reliable way?	4. Were objective, standard criteria used for measurement of the condition?	5. Were confounding factors identified?	6. Were strategies to deal with confounding factors stated?	7. Were the outcomes measured in a valid and reliable way?	8. Was appropriate statistical analysis used?	Total score (out of 8)
Abuzuluf et al. [130]	Yes (1)	Yes (1)	Yes (1)	Yes (1)	Yes (1)	Unclear (0)	Yes (1)	Yes (1)	7
Chappell et al. [26]	Yes (1)	Yes (1)	Yes (1)	Yes (1)	Unclear (0)	Unclear (0)	Yes (1)	Yes (1)	6
Lane et al. [73]	Yes (1)	Yes (1)	Yes (1)	Yes (1)	Yes (1)	Yes (1)	Yes (1)	Yes (1)	8
Lee [76]	Yes (1)	Yes (1)	Yes (1)	Yes (1)	Yes (1)	Yes (1)	Yes (1)	Yes (1)	8
Legault et al. [38]	Yes (1)	Yes (1)	Yes (1)	Yes (1)	Yes (1)	Yes (1)	Yes (1)	Yes (1)	8
Li et al. [35]	Yes (1)	Yes (1)	Yes (1)	Yes (1)	Unclear (0)	Unclear (0)	Yes (1)	Yes (1)	6
Li et al. [35]	Yes (1)	Yes (1)	Yes (1)	Yes (1)	Yes (1)	Yes (1)	Yes (1)	Yes (1)	8
Li et al. [28]	Yes (1)	Yes (1)	Yes (1)	Yes (1)	Yes (1)	Yes (1)	Yes (1)	Yes (1)	8
Magnaye et al. [87]	Yes (1)	Yes (1)	Yes (1)	Yes (1)	Yes (1)	Yes (1)	Yes (1)	Yes (1)	8
Marani et al. [117]	Yes (1)	Yes (1)	Yes (1)	Yes (1)	Unclear (0)	Unclear (0)	Yes (1)	Yes (1)	6
McCaughey et al. [61]	Yes (1)	Yes (1)	Yes (1)	Yes (1)	Unclear (0)	Unclear (0)	Yes (1)	Yes (1)	6
McCusker et al. [25]	Yes (1)	Yes (1)	Yes (1)	Yes (1)	Yes (1)	Yes (1)	Yes (1)	Yes (1)	8
Sadavoy et al. [85]	Yes (1)	Yes (1)	Unclear (0)	No (0)	Yes (1)	Yes (1)	Unclear (0)	Yes (1)	5
Sadavoy et al. [88]	Yes (1)	Yes (1)	Yes (1)	Yes (1)	Unclear (0)	Unclear (0)	Yes (1)	Yes (1)	6
Sibalija et al. [71]	Yes (1)	Yes (1)	Yes (1)	Yes (1)	Yes (1)	Yes (1)	Yes (1)	Yes (1)	8
Tam et al. [45]	Yes (1)	Yes (1)	Yes (1)	Yes (1)	Yes (1)	Yes (1)	Yes (1)	Yes (1)	8
Xiong et al. [64]	Yes (1)	Yes (1)	Yes (1)	Yes (1)	Yes (1)	Yes (1)	Yes (1)	Yes (1)	8

**TABLE 4:** Quality appraisal of mixed-methods studies (systematic review, Canada, 2020–2025).

Author(s), year	1. Were the criteria for inclusion in the sample clearly defined?	2. Were the study subjects and the setting described in detail?	3. Was the exposure measured in a valid and reliable way?	4. Were objective, standard criteria used for measurement of the condition?	5. Were confounding factors identified?	6. Were strategies to deal with confounding factors stated?	7. Were the outcomes measured in a valid and reliable way?	8. Was appropriate statistical analysis used?	9 is there congruity between the stated philosophical perspective and the research methodology?
Anderson et al. [80]	Yes (1)	Yes (1)	Yes (1)	Yes (1)	Unclear (0)	Unclear (0)	Yes (1)	Yes (1)	Yes (1)
Fox et al. [55]	Yes (1)	Yes (1)	Yes (1)	Yes (1)	No (0)	No (0)	Yes (1)	Unclear (0)	Yes (1)
Gorenko et al. [40]	Yes (1)	Yes (1)	Yes (1)	Yes (1)	Unclear (0)	Unclear (0)	Yes (1)	Yes (1)	Yes (1)
Grewal and Montgomery [131]	Yes (1)	Yes (1)	Yes (1)	Yes (1)	Unclear (0)	No (0)	Yes (1)	Yes (1)	Yes (1)
Leslie et al., [47]	Yes (1)	Yes (1)	Unclear (0)	Unclear (0)	Unclear (0)	No (0)	Unclear (0)	No (0)	Yes (1)
Schwarz et al. [137]	Yes (1)	Yes (1)	Yes (1)	Yes (1)	Unclear (0)	No (0)	Yes (1)	Yes (1)	Yes (1)
Yang et al. [72]	Yes (1)	Yes (1)	Yes (1)	Yes (1)	Unclear (0)	Unclear (0)	Yes (1)	Yes (1)	Yes (1)

10. Is there congruity between the research methodology and the research question or objectives?	11. Is there congruity between the research methodology and the methods used to collect data?	12. Is there congruity between the research methodology and the representation and analysis of data?	13. Is there congruity between the research methodology and the interpretation of results?	14. Is there a statement locating the researcher culturally or theoretically?	15. Is the influence of the researcher on the research, and vice-versa, addressed?	16. Are participants, and their voices, adequately represented?	17. Is the research ethical according to current criteria or, for recent studies, and is there evidence of ethical approval by an appropriate body?	18. Do the conclusions drawn in the research report flow from the analysis, or interpretation, of the data?	Total score (out of 18)
Yes (1)	Yes (1)	Yes (1)	Yes (1)	No (0)	No (0)	Yes (1)	Yes (1)	Yes (1)	14
Yes (1)	Yes (1)	Yes (1)	Yes (1)	Yes (1)	Yes (1)	Yes (1)	Yes (1)	Yes (1)	15
Yes (1)	Yes (1)	Yes (1)	Yes (1)	Unclear (0)	Unclear (0)	Yes (1)	Yes (1)	Yes (1)	14
Yes (1)	Yes (1)	Yes (1)	Yes (1)	Yes (1)	Yes (1)	Yes (1)	Yes (1)	Yes (1)	16
Yes (1)	Yes (1)	Yes (1)	Yes (1)	Unclear (0)	Unclear (0)	Yes (1)	Yes (1)	Yes (1)	10
Yes (1)	Yes (1)	Yes (1)	Yes (1)	Yes (1)	No (0)	Yes (1)	No (0)	Yes (1)	14
Yes (1)	Yes (1)	Yes (1)	Yes (1)	Unclear (0)	Yes (1)	Yes (1)	Yes (1)	Yes (1)	15

### Thematic Analysis and Summary of Findings

Aligned with the aim of our systematic review, five major themes and twelve corresponding sub-themes emerged: (1) Equipping caregivers to navigate the system through information access and education; (2) Addressing evolving caregiver needs via accessible and inclusive technologies; (3) Empowering caregivers in a coordinated and integrated care system to meet individual needs; (4) Supporting the emotional, social, and practical wellbeing of caregivers across the care journey; and (5) Enhancing financial and workplace supports for caregivers across diverse contexts. Refer to Supplementary Table S4 for an overview of the themes and sub-themes and supporting phrases from the studies.

#### Equipping Caregivers to Navigate the System Through Information Access and Education

Out of 83 studies reviewed, 50 highlighted the need to empower caregivers through enhanced access to information and education to help them navigate the healthcare system. Key focus areas included understanding available programs and services, accessing context-specific, high-quality, evidence-based resources, and acquiring knowledge about disease diagnosis and prognosis. Caregivers emphasized the need for improved access to clear, contextualized, and centralized information regarding available health and social services [23–47]. Many reported challenges navigating a fragmented system and expressed a desire for streamlined, easily accessible resources, particularly concerning home care, LTC, and related supports. As one caregiver remarked:

*“What is the frustration with the system, is the fact that nobody tells you what you are eligible for or what you could possibly be entitled to.”* [23] p.68

As a result, caregivers indicated feeling unprepared due to insufficient information about navigating the healthcare system and their need for more instructions on service access [27]. They voiced their frustration with the limited information about services:

*“We did not know if there was any help out there because nobody said there was any help out there.” –* Caregiver of a stroke survivor [31]. p.9

Caregivers discussed significant challenges navigating a fragmented care system and expressed a clear need for centralized access to information on available programs and services. One participant noted: *“There are so many different things, and you do not know where to go,” while* another expressed the need for *“a better roadmap of all the supports available to me*.” [47] p.5

In a Saskatchewan-based study, caregivers strongly endorsed the development of a streamlined, user-friendly website featuring instructional resources such as workshops and moderated forums, underscoring the value of a single, organized platform to access caregiver support [34].

Further, caregivers mostly sought information that was context-specific, high-quality, and evidence-based, particularly related to topics such as COVID-19, caregiving for individuals with complex conditions [45], and navigating legislation around fitness-to-drive assessments in older adults [36]. Concerning education and skill development [25, 28, 32, 34, 37, 39, 42–44, 48–63], caregivers requested anticipatory guidance and clear role description to enhance self-efficacy. For example, caregivers caring for persons with dementia highlighted the lack of adequate training during the hospital-to-home transition, noting they were unprepared to manage their care recipient’s worsening condition [44]. In another study, caregivers sought support to address feelings of inadequacy and self-doubt stemming from a lack of confidence in their caregiving abilities [51].

Caregivers of older adults with different medical conditions disclosed their need to learn more about disease diagnosis and prognosis, including clinical phases, early warning signals, and symptom management. As an instance, caregivers of persons with Alzheimer’s disease emphasized the importance of distinguishing between normal aging and early cognitive decline. As one caregiver shared:

*“Some websites say there are 7 stages, some say 3 stages … really, I just wish I knew what the stages were and what happens when.” –* Caregiver for a parent [39]. p.1054

Caregivers consistently highlighted the value of timely education and training to improve their preparedness, skills, and confidence in caregiving.

#### Addressing Caregivers’ Evolving Needs Through Accessible and Inclusive Technology

Fifteen studies reported how accessible and inclusive technology can help meet caregivers’ evolving needs [41, 45, 47, 53, 56, 57, 59, 64–69]. Caregivers identified technology as a valuable tool, particularly during the pandemic. For example, passive remote monitoring systems (e.g., in-home cameras) allowed caregivers to ensure loved ones’ safety during physical distancing restrictions [68]. Additionally, caregivers of older adults in LTC homes appreciated the use of videoconferencing to stay informed and engaged amid restrictive visitation policies [57]. However, some caregivers reported difficulty using these tools due to a lack of training and emphasized the importance of digital literacy support [67].

Caregivers also highlighted the value of two distinct types of mobile applications: general-purpose tools that indirectly support caregiving by easing everyday tasks such as medication reminders, grocery ordering, meal delivery, and transportation coordination, and caregiving-specific applications that directly support care delivery, including health monitoring tools [66]. While the former alleviate logistical burden, the latter actively empower caregivers in their care roles. Across both types, caregivers expressed a strong preference for user-friendly, cross-platform tools that integrate multiple caregiving functions:

‘‘Integration is really important, especially in caregiving so you can stay connected to what you were doing no matter what device you are using.’’ – Caregiver for a parent [66]. p.1501

There was also a strong demand for multilingual, multifunctional applications that consolidate support features into a single platform:

*“The one thing I would do better is integrate a bunch of things into one app.” –* Caregiver for a parent [66]. p.1501

Participants further suggested that technology could support safety monitoring (e.g., fall detection, emergency alerts) and called for better access to health and social services that promote and facilitate the use of digital tools in caregiving [53].

#### Empowering Caregivers in a Coordinated and Integrated Care System to Meet Individual Needs

Of the 83 studies included in this review, 56 referred to the necessity of caregivers being at the centre of a responsive, coordinated, and integrated care system. Caregivers underlined the importance of clear, coordinated, and competent communication across care settings [31, 46, 48, 52–54, 57–61, 69–78]. They underscored the need for “clear referrals”, “professional collaboration”, and efficient information transfer between healthcare providers [60]. Gaps in communication were seen as contributors to fragmented care, compromising both patient safety and quality of care [78]. Competent provider-caregiver communication was also identified as essential for building trust and promoting engagement with support services [31].

Interprofessional collaboration was viewed as critical to ensuring continuity of care. Caregivers expressed concern over poor coordination among providers, particularly during care transitions [60]. For instance, in a study involving caregivers of patients transiting from intensive care units to weaning centres, participants reported receiving inconsistent and conflicting updates from different professionals [52].

Access to interdisciplinary teams and cross-sectoral partnerships was seen as key to improving communication, care continuity, and overall caregiver experience [78]. During the COVID-19 pandemic, many caregivers reported being excluded from the care circle of older adults, underscoring the need for inclusive communication strategies during a crisis [53].

Importantly, caregivers stressed the need to be formally recognized as essential partners in collaborative care [27, 30, 32, 50, 57, 59, 60, 62, 67, 69, 74–76, 78–80]. In LTC settings, they described providing not only emotional and psychosocial support but also acting as advocates to ensure proper treatment. As one caregiver stated:

*“Families are an essential component in a resident's level of care—we are part of the team, and we have a role to play.” –* A caregiver [74]. p.5

In another study examining the experiences of caregivers to LTC residents during the pandemic, participants reported being excluded from care-related decision-making, underscoring the need for care teams that actively recognize and include caregivers as partners in the decision-making process [67].

Caregivers also advocate for supportive roles, such as community and system navigators [77] and case managers [46], to facilitate access to services and information [24, 46, 57, 72, 77, 81]. In a study examining publicly funded home care in Ontario, caregivers identified the value of a designated intermediary (a “middle-person”) to offer financial advice and bridge informational gaps between themselves, care recipients, and service providers [24]. p.1633. These findings emphasize the importance of structured support to help caregivers navigate complex care systems.

Caregivers also emphasized the significance of building a responsive and adaptable care structure [23–25, 28–30, 42, 43, 46, 48, 51, 54, 56–58, 60, 62–64, 67–69, 76–78, 81–86]. Within the context of Ontario’s Regional Frail Senior Strategy, caregivers voiced concern about frequent staff turnover, particularly in specialized geriatric services and LTC, which disrupted continuity and hindered relationship-building with healthcare providers [46]. Consistency in care personnel was seen as essential for fostering trust and improving care experiences [78].

Furthermore, the target group emphasized the need for having programs and services that take a “person-centred approach” and are based on the specific needs and circumstances of caregivers and their care recipients. A family caregiver stated:

“*The one thing that I might say is that they create programs that are kind of cookie cutter. I would [sug-gest that] during those stages, that there’s links made to services and supports that coincide with those stages. And to acknowledge and understand the dif-ferent circumstances in which people live, and the sup-ports and service they might need. Like home care, it cannot look the same in every community, because every community’s needs are different.”* [43] p.6

Another study, which aimed to investigate perspectives of caregiving among a group of Korean caregivers in Montreal, similarly underlined the significance of having person-centred resources that consider the unique needs of caregivers [83]. The participants in this study acknowledged that despite their ability to navigate the Canadian healthcare system, they found language proficiency as a barrier to accessing and using the services:

*“It might be difficult for my parents and myself to find resources in Montreal. As you know, French proficiency affects access to health services here.” –* Caregiver for a parent [83] p.4889

Hence, caregivers emphasized the importance of a responsive care system that is attuned to individuals’ background, preferences, and social context to provide meaningful and personalized support.

In addition, they highlighted the need for flexible, needs-based visitation policies in times of crisis [43–45, 57, 67, 69, 75, 79]. For instance, caregivers noted that inconsistent or last-minute updates from LTC homes regarding visitation during the pandemic often conflicted with work and childcare responsibilities, making visits difficult to arrange [79]. Restrictions on visiting loved ones in LTC or palliative care settings were cited as one of the caregivers’ most distressing experiences, highlighting the need for policies that balance health measures with the preservation of essential social bonds [45]. Some caregivers pointed to successful examples of safe, regular visits, reinforcing the critical role of visitation in maintaining connection and care. As such, participants advocated for more adaptable visitation policies that support continuity of care and emotional wellbeing while observing necessary safety protocols:

*“Yeah, I’ve been visiting regularly for the last two weeks, three times a week, with the mask, the goggles, the gown. Everything is well, everything is A-1 as a precaution. […] We had an information course, for an hour and a half [on preventive measures].”* – Caregiver for a friend [57] p.251

Thus, caregivers suggested that visitation policies should be designed with greater flexibility, ensuring that caregivers can maintain meaningful connections with loved ones while adhering to safety protocols.

#### Supporting the Emotional, Social, and Practical Wellbeing of Caregivers Across the Care Journey

Fifty-five [55] out of the 83 studies discussed the need for caregivers to support their emotional, social and practical wellbeing. As part of this need, caregivers stressed the importance of receiving emotional and mental health support from providers [23, 26, 27, 29, 34–41, 44, 45, 48–54, 56, 57, 59, 61, 69, 70, 73, 74, 76, 80–82, 85, 87, 88]. A family caregiver noted:

*“I mean, the physician does not even ask: how are you [the caregiver] managing? Who’s doing your meals? Who’s doing the shopping? They do not ask any of that.”* [59] p.225

In a co-design study exploring caregivers’ needs and goals, participants prioritized their physical, mental, and emotional health over direct care-related responsibilities, indicating the significant burden of caregiving and the need for systemic support [87]. Employed healthcare providers of people with dementia, in particular, identified “dementia demands” and the unpredictability of behavioural symptoms as major sources of emotional strain, highlighting the urgent need for mental health resources [88]. Moreover, caregivers of LTC residents underlined the emotional toll of the pandemic-related restrictions, citing that prolonged separation intensified distress for both themselves and care recipients [69]. They advocated for future public health responses to incorporate emotional and psychological support for caregivers. Collectively, these findings underscore caregivers’ call for healthcare and continuing care policies that explicitly prioritize their emotional and mental wellbeing.

Caregivers also expressed a great desire for social connections and community-based support networks [26, 31, 35, 37, 40, 41, 43–46, 48, 49, 53, 56, 57, 59, 60, 62, 65, 70, 73, 78, 80–83, 87], particularly in managing grief and bereavement following the death of a loved one [70]. They also underscored the value of peer support and community-based support networks. In a study on healthcare transitions for persons living with dementia, caregivers highlighted how sharing “parallel experiences” with peers helped them anticipate challenges and advocate more effectively for needed support [60]. These findings emphasize the need to strengthen support networks and encourage community engagement to improve caregiver resilience.

Furthermore, caregivers consistently reported a need for practical relief through accessible respite services [23, 27, 29–32, 34, 37, 39, 41, 42, 44, 46, 48–50, 53, 56–58, 62, 72, 80–82, 87, 88]. In a Saskatchewan-based study, caregivers identified respite and self-care activities, such as taking a few hours off weekly, a top priority for maintaining their wellbeing [34]. Preferred options included in-home respite, adult day programs, and structured self-care support.

Additionally, caregivers expressed a need for assistance with daily physical care tasks (e.g., bathing, toileting, and feeding) and instrumental activities (e.g., transportation, shopping, home maintenance, managing medication). One family caregiver of a stroke survivor stated:

“*I wish I could get a service, a drive to [husband]’s day program, but apparently we’re in a dead zone or something.”* [31] p.7

These needs became especially urgent during the COVID-19 pandemic, when many essential caregiving supports, including respite care, home care, and day programs, were suddenly discontinued, leaving caregivers to shoulder significant physical and emotional burdens alone:

*“[Before] COVID hit, I had help every day . . . we had the day programs twice a week, I had hired a driver, I had a couple of days of respite, I also had a housekeeper to do the hard stuff, I’ve had that for a few years because I’m getting older too. And of course all that disappeared, so I was totally on my own . . . I was doing a lot of yard work and I’m pretty much doing that all myself at this point, doing housework and big jobs I have not had to do for probably about eight or so years.” – Caregiver for a person with dementia.* [29] p.224

So, there is a need for accessible relief programs and adapted respite services to support caregivers effectively.

#### Enhancing Financial and Workplace Supports for Caregivers Across Diverse Contexts

Among the 83 studies reviewed, 31 emphasized the need for enhancing financial and workplace supports for caregivers across diverse contexts. Caregivers consistently highlighted the material and financial burdens of caregiving, citing challenges such as job loss, reduced income, out-of-pocket costs for medical equipment, hiring personal support workers, and funding home repairs and improvements [23, 24, 26, 27, 30–32, 35, 37, 38, 44, 48, 51, 53, 56, 57, 73, 80, 85, 87–90]. One caregiver of a parent with dementia in rural Saskatchewan stated that the inadequacy of current financial relief:

“*Very limited tax breaks for caregivers. It costs a lot of money as well as most requiring care have a limited income”.* [51] p.5

Many called for targeted financial assistance, including tax credits and government grants. The compounded financial strain of “double caregiving”—simultaneously caring for aging parents and dependent children—was also identified as a critical issue [51]. These challenges were further exacerbated during the COVID-19 pandemic, when heightened care demands impeded caregivers’ ability to seek or maintain employment:

*“Was trying to look for work and having interviews, but due to the increase of care needed, and COVID-19, I am unable to find work and not qualified for any government financial help which adds to the current problems.” –* A caregiver living with the care recipient [80] p.9

Finally, caregivers stressed the need for caregiver-friendly workplaces and flexible employment policies [27, 37, 38, 42, 65, 85, 87, 88, 90–92]. Key supports included flexible scheduling, access to workplace resources, and accommodations tailored to specific groups, such as transnational caregivers (employed caregivers caring for loved ones across international borders) and double- or triple-duty carers (those simultaneously engaged in paid care work and unpaid caregiving responsibilities for relatives or friends across multiple domains).

Flexible employment policies were underlined by the participants in a study that investigated experiences of caregivers in working and caregiving from home during the COVID-19 pandemic:

*“For me, I can honestly say that the COVID lockdown helped. Because with the company, allowing us to work from home, that really helps to be able to spread out one's workday, and also enable someone to be able to provide support, you know, whether physically or remotely in some way needed.”* – Caregiver for a parent [91] p.165

Caregivers referred to flexibility and work-life balance as critical work-related demands. For instance, a transnational caregiver who lives and works full-time in Canada while providing care to a family member in the country of birth, mentioned:

“*Getting time off can sometimes be really challenging, like a day or two days; even as a part-time worker, there’s almost this hold on your time. It depends where you work.”* [65] p.5

Accordingly, caregivers asked for caregiver-friendly workplace policies to support employees balancing paid work and caregiving responsibilities.

## Discussion

This systematic review presents the support needs of caregivers for older adults in Canada, highlighting significant gaps in the current landscape in terms of the availability, accessibility, and coordination of services. Caregivers emphasized the need for clear, contextualized, and centralized information on health and social services, particularly home care and LTC. In line with prior research [93, 94], this review highlights the persistent challenges caregivers face in accessing appropriate resources and navigating a fragmented and complex healthcare system. These findings are further reinforced by qualitative studies from the United States (U.S.) and Europe, which also document barriers to accessing timely, accurate, and relevant information [95, 96], while also noting the potential of psychoeducation programs and improved dissemination strategies to enhance caregiver wellbeing [95, 96]. Furthermore, caregivers stressed the importance of education, anticipatory guidance, and context-specific knowledge—particularly related to dementia, public health crises, such as COVID-19, and navigating complex legal processes.

A 2021 study [97] investigating the needs of caregivers for individuals with dementia found that caregivers often lack adequate information about the expected progression of the disease, available treatment options, and services tailored to both patients and caregivers. These informational needs were frequently reported as either unmet or only partially met [97]. Similarly, an analysis of dementia care in rural and remote communities revealed a widespread need among both healthcare providers and caregivers for ongoing education related to dementia symptoms, diagnosis, and care management [98]. Consistently, evidence shows that education, training, and mentorship not only reduce caregiver stress but also improve care quality [99–101].

Our findings underscore the emerging role of technology as a vital communication tool for caregivers, particularly during the pandemic. Consistent with international studies and reviews [102, 103], caregivers, many already experiencing substantial burden, reported barriers related to cost, device delivery and installation, digital access inequities (including limited internet connectivity and personal devices), and the self-directed nature of some technological solutions. Despite limited prior exposure to digital platforms, caregivers recognized the potential value of technology and expressed willingness to adopt accessible, user-friendly solutions tailored to their needs [97]. Supporting this finding, a scoping review of internet-based interventions found that caregivers used online platforms to reduce social isolation and access resources, demonstrating the potential of digital tools as accessible and cost-effective support mechanisms [103]. Furthermore, a pilot study in Germany involving community-dwelling dementia caregiving dyads (persons with dementia and their caregivers) showed that “social technology” facilitated meaningful interactions and strengthened emotional connection during periods of physical distancing [104].

This systematic review amplifies caregivers’ need for enhanced communication and coordination in care delivery, calling for improved interprofessional and inter-organizational collaboration, as well as the formal recognition of caregivers as essential partners in care planning and delivery. These findings align with a growing body of literature that advocates for effective collaboration as a cornerstone of patient-centred care for older adults and their families [105, 106], and highlights caregivers’ role in ensuring quality outcomes [107–110]. For instance, Elliott and colleagues (2018) conducted co-design workshops involving patients, caregivers, and healthcare providers (HCPs) to collaboratively identify key priorities for improving family engagement, with communication and caregiver involvement during care transitions emerging as key areas for improvement [107]. During the pandemic, patients, caregivers, and HCPs across Canadian acute care settings further underscored caregivers’ critical role, advocating for their recognition as essential members of the care team [108]. Moreover, our findings point to the value of supportive roles such as system navigators and case managers in empowering caregivers and addressing persistent gaps in healthcare navigation. While Luke et al. focused on caregivers of children and youth, their findings highlight the effectiveness of patient navigation programs in supporting caregivers to navigate complex systems, suggesting the transferability of such models to the context of caregiving for older adult [111]. Complementary research identifies navigators and case managers as vital to transitions by coordinating services, ensuring timely access to care, and facilitating continuity [112].

Our review highlights caregivers’ need for a responsive and adaptable care system that prioritizes patient-centred care, consistent staffing, and HCP training that reflects caregivers’ roles and needs. Studies developing a competency framework for HCP education emphasize the importance of consistent, person-centred approaches to care [109]. Training programs focused on caregiver-centred competencies have been shown to improve caregiver support and enhance responsiveness in care delivery [113]. Caregivers also stressed the importance of staff stability in order to enhance continuity of care and build trustworthy relationships with providers [114]. The COVID-19 pandemic further exposed systemic rigidity, particularly regarding visitation policies in Canadian healthcare settings such as LTC homes, hospitals. A qualitative study by McMillan et al. found that restrictive visiting policies in Canadian acute care settings during the pandemic and found that such policies disrupted essential caregiving roles in providing emotional support, advocacy, and communication, prompting calls for more individualized, person- and family-centred care principles [108]. Caregivers of LTC residents also noted the significant emotional distress and trauma due to prolonged separation, advocating for collaborative and flexible policy changes that balance infection control with the psychosocial needs of residents and their families [110, 115].

This review’s findings further underscore the need of addressing caregivers’ mental health and emotional wellbeing, as well as strengthening community networks and peer support to reduce feelings of loneliness, isolation and enhance coping. Data from the Canadian Longitudinal Study on Aging indicate that greater social participation and support are linked to lower depression levels among caregivers, underscoring the protective impact of social engagement in caregiver mental health [71]. Beyond emotional support, caregivers stressed the need for respite and practical support, including help with activities of daily living (ADLs). Research has shown the value of flexible, culturally sensitive, and well-communicated respite choices. For example, in a U.S. study, Gaugler et al. found that adult day programs, as a form of respite care, not only supported caregivers in sustaining their role but also enabled them to reorganize their time, reducing caregiving demands related to ADLs and allowing older adults to remain in the community longer [116].

Lastly, this review highlights the pervasive financial strain and employment compromises faced by caregivers for older adults in Canada, with a disproportionate burden falling on women [6]. The gendered nature of caregiving has far-reaching implications for caregivers’ economic security, career trajectories, and overall wellbeing. These burdens stem not only from lost income but also from unpaid leave and substantial out-of-pocket expenses for home modifications, medical equipment, respite care, transportation, and medications [117–120]. To sustain their caregiving role, caregivers require workplace flexibility, employment protections, and financial assistance. The evidence aligns with broader research indicating that over one-quarter of Canadian caregivers make significant career adjustments—such as reducing work hours, forgoing promotions, or leaving the workforce—to meet caregiving demands [121–123]. This review reinforces the urgent need for caregiver-friendly workplace policies, including flexible schedules, paid or unpaid leave, telework options, and supportive supervisory practices. As noted by Li and Lee (2020), such accommodations are essential to helping caregivers maintain employment while reducing the risk of work–family conflict [123].

### Strengths and Limitations

This systematic review offers several notable strengths and contributions. Unlike prior reviews that focus on caregivers of older adults with specific chronic conditions—such as dementia, cancer, or palliative care—this review adopts a broader perspective, examining the support needs of caregivers regardless of diagnosis. This inclusive approach captures common challenges across caregiving contexts, such as emotional strain, financial hardship, social isolation, lack of respite, and difficulties navigating health systems. By identifying these cross-cutting needs, the findings can inform system-level and policy interventions with broad applicability. This general focus also promotes equity by recognizing diverse caregiving experiences, including those that begin prior to a formal diagnosis or involve age-related decline rather than a defined illness, thereby supporting the development of more inclusive, proactive, and responsive caregiver support services.

Another strength lies in its inclusion of studies across a range of care settings, including community, LTC facilities, hospitals, and transitional care, capturing the evolving nature of caregiver needs across the care continuum. This comprehensive scope enables for both a nuanced understanding of caregiver needs and identification of setting-specific and universal challenges, informing the design of both targeted and broad-based interventions. Importantly, by including studies conducted during and after the COVID-19 pandemic (2020–2025), this review reflects recent caregiving realities marked by heightened stress, service disruptions, increased isolation, and expanded responsibilities. These insights are especially timely for shaping post-pandemic recovery efforts.

Additionally, the review adopts a multidimensional perspective, examining the full spectrum of caregiver needs—emotional, social, practical, and systemic—rather than limiting the focus to individual domains. This holistic view highlights the interconnected nature of support needs and reinforces the importance of integrated, multi-component interventions.

While this review offers several strengths, a few limitations warrant consideration. The exclusion of grey literature, such as government and community organization reports, may have omitted relevant findings from non-academic sources, including recent policy evaluations and grassroots perspectives. Limiting the review to English-language publications may also have excluded important perspectives from Francophone or bilingual regions of Canada, like Quebec, potentially missing culturally and linguistically distinct experiences. Moreover, while the Canada-specific focus enhances national relevance, it may constrain the generalizability of findings to other healthcare systems. Nonetheless, the deliberate inclusion of peer-reviewed, Canadian-based, caregiver-centred studies offers a robust, evidence base to inform policy, service design, and future research. Further investigation into caregiver characteristics could yield more nuanced, context-specific insights to better address their evolving needs.

### Implications

The findings of this systematic review carry important implications for policy, practice, and research. At the policy and systems level, they underscore the urgent need for coordinated, caregiver-centred frameworks that enable equitable access to clear, centralized information and navigation support across care settings. Strategic investment in dedicated caregiver navigation roles, such as case managers or system navigators, is warranted to mitigate or reduce service fragmentation and enhance caregivers’ ability to access and coordinate services effectively. Caregivers must also be formally recognized as essential care partners, with their expertise meaningfully integrated into care planning and decision-making, particularly within long-term care and transitional care contexts. Despite caregiver recognition legislation and supportive initiatives across several Canadian jurisdictions [124–128], caregivers remain insufficiently embedded within health system processes. Policy frameworks must therefore move beyond symbolic recognition to explicitly affirm caregivers’ roles and rights and address their financial vulnerability through targeted income and tax-based supports, such as the Canada Caregiver Credit [129]. Persistent barriers, including restrictive eligibility criteria, limited benefit duration and scope, administrative complexity, and low program awareness, continue to limit the effectiveness of existing federal supports, such as Employment Insurance (EI) caregiving benefits [13]. Addressing these gaps will require coordinated federal leadership, sustained caregiver engagement, and modernization of caregiving policies, alongside continued investment in successful aging- and dementia-focused strategies (e.g., Canada’s National Dementia Strategy) that prioritize the needs of both care recipients and caregivers through education, navigation tools, and community-based programs [14].

From a service design perspective, health and social service providers should prioritize flexible, person- and community-centred supports with demonstrated impact, such as the Pan-Canadian Social Prescribing initiatives, which connect caregivers to community-based resources that address social, emotional, and practical needs through a strengths-based approach [14]. Supports must be responsive to caregivers’ diverse cultural, linguistic, and geographic contexts, as standardized, one-size-fits-all models fail to reflect the heterogeneity of caregiving experiences. Instead, adaptable and individualized programs are needed to promote equitable and meaningful support. Strengthening digital infrastructure is also critical, as caregivers require access to user-friendly, multilingual, and multifunctional platforms, alongside training and ongoing support to build digital literacy as virtual care becomes more prevalent. Finally, investment in provider education is essential to build caregiver-centred competencies, including the skills needed to effectively engage caregivers and support their emotional, practical, and informational needs across the care continuum.

In terms of research implications, future studies should explore the context-specific experiences of caregivers across diverse regions, cultural backgrounds, and caregiving contexts—such as rural vs. urban settings, immigrant populations, and racialized communities. There is a pressing need for intervention studies employing co-design approaches with caregivers from heterogeneous backgrounds to develop and evaluate multidimensional supports addressing education, mental health, financial assistance, and social connectedness. Additionally, longitudinal research is essential to understand how caregiver needs evolve over time and during key care transitions (e.g., hospital to home, community to LTC), and to inform the development of adaptive, responsive support systems.

### Conclusion

This systematic review highlights the complex and evolving support needs of caregivers for older adults in Canada, including informational, emotional, technological, practical, and financial aspects. Caregivers consistently emphasized the importance of accessible, clear information, and the need for inclusive and integrated care systems, and their recognition as essential partners in the delivery of care. Addressing these priorities requires the development of person-centred, flexible, and culturally responsive policies and services. By focusing on caregivers’ perspectives and voices, this review provides critical insights to guide the development of caregiver-informed interventions, strengthen systemic supports, and inform future research aimed at improving caregiver wellbeing and the overall quality of care for older adults.

## Footnotes

1 Some studies were conducted in multiple provinces, so they were counted more than once.
