# Correction: Unveiling the Hidden Challenges: A Systematic Review of Self-Identified Support Needs of Caregivers for Older Adults in Canada

**DOI:** 10.3389/phrs.2026.1609718

**Published:** 2026-03-17

**Authors:** Sheila A. Boamah, Hoda Herati, Farzana Akter, Farinaz Havaei, Marie-Lee Yous, Sharon Kaasalainen

**Affiliations:** 1 McMaster University School of Nursing, Hamilton, ON, Canada; 2 The University of British Columbia, Vancouver, BC, Canada

**Keywords:** Canada, informal care, older adults, self-identified caregivers, support needs

Figure 1 in the published version appears to be improperly formatted. The correctly formatted version of appears below.

**FIGURE 1:**
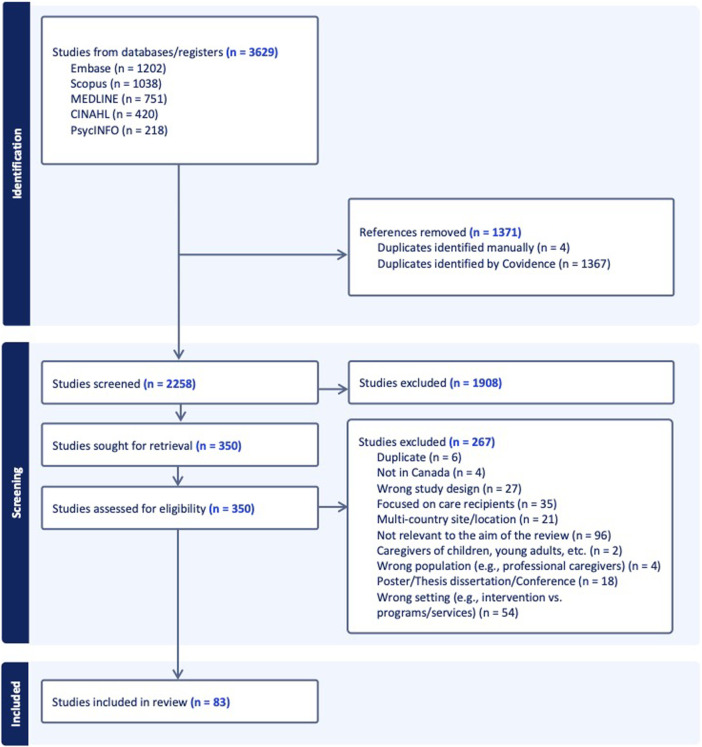
PRISMA flowchart diagram of the selection process (Canada, 2025).

In the first published version of the article there was an error. There were residual blinded statements that were not removed prior to publication. These occurred in the **Methods** section, sub-sections: *Selection Process*, *Data Collection/Extraction Process,* and *Quality Appraisal (Assessment of Risk of Bias)*, paragraph 1. Each of these sections contained the statement “(blinded for peer-review)”. These placeholders have been removed.

A correction has been made to **Methods,** *Selection Process*. “Three reviewers (HH, FA, MA) independently screened the titles and abstracts of 2258 studies based on predetermined inclusion and exclusion criteria. As a result, 1908 studies were excluded, and 350 studies were retrieved for full-text screening, which was conducted independently by the three researchers. Discrepancies were resolved through discussion, when unresolved cases at the full-text stage adjudicated by the lead researcher (SB).”

A correction has been made to **Methods,** *Data Collection/Extraction Process*. “Data extraction was carried out independently by at least two reviewers using Covidence, a widely utilized platform for screening and data extraction in literature reviews.”

A correction has been made to **Methods,** *Quality Appraisal (Assessment of Risk of Bias)*, Paragraph 1. “The methodological quality of the included qualitative and quantitative studies was assessed by two reviewers (HH, FA) independently using the Joanna Briggs Institute (JBI) critical appraisal tools relevant to each study design (17,18).”

The original article has been updated.

