# Supplementary material for: Unveiling the Hidden Challenges: A Systematic Review of Self-Identified Caregiver Support Needs for Older Adults in Canada
Source: Public Health Rev. 2026 Feb 26;47:1609117. doi: 10.3389/phrs.2026.1609117 (PMC12979237; doi:10.3389/phrs.2026.1609117)
Supplement: Supplementary file 1 [file Table1.docx]

Supplementary Table S1—Search strategy for database

| **Database** | **Search Strategy** | **Results** |
| --- | --- | --- |
| Embase | 1 exp caregiver/  2 informal caregiver*.mp.  3 unpaid caregiver*.mp.  4 carer*.mp.  5 family caregiver*.mp.  6 care partner*.mp.  7 support*.mp.  8 service*.mp.  9 intervention*.mp.  10 resource*.mp.  11 caregiver support.mp. or exp caregiver support/  12 exp Canada/ or Canada.mp.  13 1 or 2 or 3 or 4 or 5 or 6  14 7 or 8 or 9 or 10 or 11  15 12 and 13 and 14  16 limit 15 to (english language and yr="2020 - current") | 1202 |
| MEDLINE | **1** exp Caregivers  **2** informal caregiver*.mp.  **3** unpaid caregiver*.mp.  4 carer*.mp.  5 family caregiver*.mp.  6 care partner*.mp.  7 1 or 2 or 3 or 4 or 5 or 6  8 exp Caregivers/ or Caregiver support.mp.  9 support*.mp.  10 service*.mp.  11 intervention*.mp.  12 Resource*.mp.  13 caregiver support*.mp.  14 8 or 9 or 10 or 11or 12 or 13  15 exp Canada/  16 Canada*.mp.  17 15 or 16  18 7 and 14 and 17  19 limit 18 to (english language and yr="2020 - current" | 751 |
| PsycINFO | 1 exp Caregivers  2 informal caregiver*.mp.  3 unpaid caregiver*.mp.  4 carer*.mp.  5 family caregiver*.mp.  6 care partner*.mp.  7 1 or 2 or 3 or 4 or 5 or 6  8 exp Caregivers/ or Caregiver support.mp.  9 support*.mp.  10 service*.mp.  11 intervention*.mp.  12 Resource*.mp.  13 caregiver support*.mp.  14 8 or 9 or 10 or 11or 12 or 13  15 Canada.mp.  16 7 AND 14 AND 15  17 limit 16 to (English language and yr="2020 - current") | 218 |
| CINAHL | S1 (MH "Caregivers")  S2 "informal caregiver*"  S3 "unpaid caregiver*"  S4 "carer*"  S5 "family caregiver*"  S6 "care partner*"  S7 S1 OR S2 OR S3 OR S4 OR S5 OR S6  S8 (MH "Caregiver Support")  S9 "service*"  S10 "support*"  S11 Resource*  S12 " intervention*"  S13 caregiver support*  S14 S8 OR S9 OR S10 OR S15 OR S12 OR S13  S16 (MH "Canada")  S17 “CANADA”  S18 S16 OR S17  S19 S7 AND S14 AND S18  Limiters – Publication Date: 20240101-20250630; English Language | 420 |
| Scopus | ( TITLE-ABS-KEY ( caregiver ) OR TITLE-ABS-KEY ( informal AND caregivers ) OR TITLE-ABS-KEY ( unpaid AND caregivers ) OR TITLE-ABS-KEY ( carers ) OR TITLE-ABS-KEY ( family AND caregivers ) OR TITLE-ABS-KEY ( care AND partners ) AND TITLE-ABS-KEY ( caregiver AND support ) OR TITLE-ABS-KEY ( support ) OR TITLE-ABS-KEY ( services ) OR TITLE-ABS-KEY ( interventions ) OR TITLE-ABS-KEY ( resources ) AND TITLE-ABS-KEY ( canada ) AND NOT TITLE-ABS-KEY ( child* ) AND NOT TITLE-ABS-KEY ( infants ) AND NOT TITLE-ABS-KEY ( adolescents ) AND NOT TITLE-ABS-KEY ( youths ) ) AND PUBYEAR > 2019 AND PUBYEAR < 2026 AND ( LIMIT-TO ( LANGUAGE , "english" ) ) AND ( LIMIT-TO ( DOCTYPE , "ar" ) ) | 1038 |
|  | Total | 3,629 |
