# Supplementary material for: Unveiling the Hidden Challenges: A Systematic Review of Self-Identified Caregiver Support Needs for Older Adults in Canada
Source: Public Health Rev. 2026 Feb 26;47:1609117. doi: 10.3389/phrs.2026.1609117 (PMC12979237; doi:10.3389/phrs.2026.1609117)
Supplement: Supplementary file 2 [file Table2.docx]

Supplementary Table S2—Inclusion and exclusion criteria

| Criteria | Inclusion | Exclusion |
| --- | --- | --- |
| Population, Exposure, Outcome (PEO) Component | *Population*: unpaid family/friend caregivers of older adults (60+) in Canada  *Exposure*: studies on caregivers’ experiences of providing care to older adults with chronic conditions (even if age was not specified to avoid omitting significant findings pertinent to our target population)  *Outcome*: identified support needs (emotional, financial, practical, etc.) of caregivers | Studies primarily focused on professional or paid caregivers, or caregivers of children/young adults |
| Publication Type | Peer-reviewed journal articles | Grey literature (e.g., theses, dissertations, government reports), reviews, meta-analyses, poster/conference abstracts |
| Geographic Focus | Studies focused exclusively on Canada | Studies focused on countries other than Canada, or multi-country studies that included Canada |
| Time Frame | Articles published between January 2020 and July 2024 | Articles published outside the specified date range |
| Language | English | Articles published in languages other than English |
