# Supplementary material for: Unveiling the Hidden Challenges: A Systematic Review of Self-Identified Caregiver Support Needs for Older Adults in Canada
Source: Public Health Rev. 2026 Feb 26;47:1609117. doi: 10.3389/phrs.2026.1609117 (PMC12979237; doi:10.3389/phrs.2026.1609117)
Supplement: Supplementary file 3 [file Table3.docx]

**Supplementary Table S3—Summary of Included Studies**

|  | Author, Year | Province(s) | Aim of Study | Study Design (methods) | Sample Population | Sub-group Size (if applicable) | Total (N) | Caregivers Age Range | Caregivers Mean Age | Care Recipients Age Range | Care Recipients Mean Age | Care Recipient Condition | Support Needs |
| --- | --- | --- | --- | --- | --- | --- | --- | --- | --- | --- | --- | --- | --- |
|  | Abuzuluf et al., 2025 | Ontario | Identify the unique needs of lung cancer patients and their caregivers | Cross-sectional (Self-report survey) | Patients with lung cancer (PLC) and FCs | PLC (n=103), caregivers (n=96) | N = 199 | 23 - 85 | 55 | 33 - 91 | 70 | Lung cancer | - Cancer-related medical information (e.g., treatment options, complications of lung cancer, prognosis, etc.),  - Information through one-on-one counselling with HCPs, websites, pamphlets, and online audio. |
|  | Anderson et al., 2021 | Alberta | Better understand the impact of the COVID-19 pandemic on anxiety, loneliness, and care work of family caregivers | Mixed-method - Retrospective design as the data were collected before the pandemic began and four months after the pandemic was declared (Online survey with two open-ended qualitative questions) | Family caregivers |  | N= 604 | 15 - 75+ |  | Birth - 75+ |  | Disabilities (physical or cognitive impairments), Chronic conditions (e.g., cardiovascular diseases, diabetes), Dementia, Frailty due to aging, Mental illness, Terminal or serious illnesses, Drug or alcohol dependency | - Emotional and mental health support,  - Inclusion in care decisions,  - Social connection,  - Respite and home care services,  - Financial support |
|  | Ashbourne et al., 2021 | Ontario | Explore the care transition experiences of individuals living with dementia and their caregivers and to use these perspectives to develop a theoretical framework outlining the factors affecting health care transitions. | Qualitative (Individual and dyad interviews) | 1. Individuals with dementia, 2. Informal caregivers | Individuals with dementia (n=12), Informal caregivers (n=29) | N= 41 | 56 - 80 | 69 | 58 - 97 | 78 | Dementia | - Caregiver's viewpoints being respected,  - Professional collaboration,  - Provider consistency,  - Peer support, - Patient and caregiver education,  - Person-centred care |
|  | Barber et al., 2022 | Nova Scotia | Identify factors that contribute to older adult patients being assessed as requiring long-term care when they could potentially return home with enhanced supports. | Qualitative - Retrospective case study design (In-person interviews) | 1. Older adult patients, 2. Patients' family caregivers, 3. Healthcare professionals including nurse managers and community care coordinator | Older adult patient (n=1), Patients' family caregivers (n=4), Healthcare professionals (n=4) | N= 9 (nine interviews focused on three patient cases) | Not reported |  | Not reported |  | Not reported | - Timely information about home care services,  - Knowledge to navigate the resources required to continue caring for older adults at home. |
|  | Bélanger-Dibblee et al., 2023 | Quebec | Explore the needs and experience of caregivers of patients with Behavioral and psychological symptoms of dementia (BPSD) living in centre dâ€™hÂ´ebergement de soins de longue durÂ´ee (CHSLDs), as well as the solutions they suggest to better manage BPSD in CHSLDs | Qualitative - Interpretive descriptive (Focus groups) | Family caregivers |  | N= 32 | 47-82 |  | Not reported |  | Dementia | - Smoother transition from home to institutional care,  - Basic training on knowledge about BPSD,  - Adapted approach to BPSD by healthcare professionals,  - Improvement in communication,  - Define the role of the caregiver in partnership with the healthcare team,  - Respite care. |
|  | Boamah et al., 2024 | Ontario | Explore the lived experiences of caregivers of people living in long-term care (LTC) homes during the initial phases of the COVID-19 pandemic and potential supports and resources needed to improve caregivers' quality of life. | Qualitative - Interpretive approach (Photovoice and Focus groups) | Family caregivers of older adults living in Ontario LTC homes |  | N= 6 | 18+ |  | Not reported |  | Not reported | - Clear communication channels with LTC homes,  - Inclusion of FCs in policy and decision-making process of care plans,  - Clear and consistent messaging related to LTC policies during the pandemic (e.g., visitor rules),  - Flexible rules considering the needs of LTC residents,  - Designate family caregivers as essentials |
|  | Boamah et al., 2024 | Ontario | Understand how measures to manage COVID-19 pandemic affected informal caregivers of people living in LTC homes in Ontario. Strategies to increase socialization and promote social connection during and post-COVID-19 were also explored. | Qualitative - Interpretive descriptive (Photovoice and virtual focus groups) | Family caregivers of older adults living in LTC homes in Ontario |  | N= 6 | 18+ |  | Not reported |  | Not reported | - Considering caregivers as 'essentials' rather than 'visitors',  - Consistent and clear visitor rules/restriction during the pandemic,  - Person-centred rules,  - Training/assistance by the LTC homes in using technology for social connections (e.g., videoconferencing),  - Strategies to communicate with residents with sensory and cognitive impairments, considering their needs,  - Virtual support groups for caregivers |
|  | Bourbonnais et al., 2024 | Quebec | Describe the experiences of family care partners of older persons living in long-term care homes (LTCH) and solicit their recommendations for supportive actions | Qualitative - Critical ethnography (Interviews) | Care partners who cared or had cared for an older person living in an LTC homes in Quebec during the COVID-19 pandemic |  | N= 24 |  | 62.8 | 65+ | 87.8 | Neurocognitive disorder, stroke, etc. | - Take actions to help care partners (e.g., care partners as essential in LTCHs, supervise them during a crisis rather than ban visits, train care partners to assist older persons, provide psychological support to care partners, encourage care partners to seek support from family/friends),  - Take actions for quality care (e.g., increase number of staff, stable care teams, more qualified and well-trained professionals, develop a crisis management plan, involve care partners in crisis care, express empathy and support for bereaved care partners),  - Take actions to communicate with care partners (e.g., be proactively informed and reassured, identify designated persons to communicate regularly with care partners, frequent and regular videoconferencing meetings/sharing video and photos with care partners),  - Take actions to give care partners access (e.g., train care partners to apply preventive measures),  - Take actions to understand the reality (e.g., identify a spokesperson, to inform the government about what was happening in LTCHs, identify ways to allow care partners to temporarily care for the older person in their home during a crisis, including offering home support and financial support) |
|  | Chappell et al., 2023 | Canada | Explore the intersectionality of gender, relationship to the care recipient, and caregiving demands in relation to the wellbeing of family caregivers. | Cross-sectional (secondary data analysis) | Family caregivers (Spousal and adult child) | Less-intensive caregivers (providing less than 36 hours/week), n = 5,364, Very-intensive caregivers (providing 36+ hours/week), n=630 | N= 5,994 |  | Less-intensive caregivers (59.5) and very-intensive caregivers (62.5) | Not reported |  | Not reported | - Social support (e.g., social participation in community-based activities),  - Financial assistance,  - Emotional support and companionship based on the care demand to maintain well-being of caregivers  -Information support |
|  | Chu et al., 2022 | Ontario and British Columbia | Explore the essential family caregivers trauma of being locked out of long-term care homes (LTCHs) and unable to visit their loved ones in-person during COVID-19. | Qualitative (Focus groups) | Essential family caregivers of LTC home residents |  | N= 30 | 35 - 65+ |  | Not reported |  | Not reported | - Collaborative LTC health system, so caregivers to be: 1) consistently involved in health policy decision-making, 2) updated on care practices/plan, 3) supported socially and emotionally,  - Revision of provincial/federal legislation regarding visitation restrictions of family caregivers. |
|  | Conklin et al., 2024 | Ontario | Understand the nature of the care provided by Ontario's family caregivers of older adults living in LTC homes during the pandemic lockdown. | Qualitative (a single open-ended question in a survey) | Family caregivers of residences in LTC homes during the COVID-19 pandemic |  | N = 192 (119 answsered the open-ended question in the survey) | 26 - 80+ |  | Not reported |  | Not reported | - Considering families as a part of the LTC care team,  - Improvement in LTC homes deficiencies and shortcomings, including better communication and transparency,  - Mental health support,  - Peer counselling |
|  | Cooper et al., 2020 | Manitoba | Explore the experiences of Metis caregivers providing care for older Metis adults. | Qualitative (Interviews and focus groups) | 1. Metis family caregivers, 2. Metis Elders, 3. Formal caregivers |  | N= 98 | 19+ |  | Not reported but the care recipents were identified as Elders |  | Not reported | - Culturally relevant care services,  - Greater involvement in healthcare decisions,  - Clearer information on available resources,  - Financial support |
|  | Cruz et al., 2024 | Ontario | Explore the lived experiences of caregivers who are providing care to an adult family member who is living with a mental illness in Windsor-Essex County, Ontario. | Qualitative - Descriptive (Interviews) | Informal caregivers providing care to persons living with mental illness and/or addiction |  | N= 21 | 27 - 72 |  | Not reported, but the care recipients included children, partners and siblings |  | Anxiety disorders, bipolar disorder, depression, schizophrenia, and substance use disorders | - Emotional and psychological support,  Financial and employment support,  - Access to health and social services,  - Recognition as essential care partners,  - Respite and self-care opportunities |
|  | Dale et al., 2020 | Ontario | Explore potentially modifiable support needs and care processes of importance to family caregivers of patients requiring prolonged mechanical ventilation and transition from the intensive care unit to a specialised weaning centre | Qualitative - longitudinal descriptive (Serial interviews) | Family caregivers of patients requiring prolonged mechanical ventilation and admission to a specialised weaning centre |  | N= 18 | 23 - 83 | 47 | 34 - 93 | 65 | Mechanical ventilation | - Informational support,  - Emotional support,  - Provide training to caregivers |
|  | Ding et al., 2022 | Canada | Investigate the changing meanings of home as a place for care during the COVID-19 pandemic. | Qualitative (Interviews) | Informal caregivers engaged in a workplace |  | N= 5 | 35 - 65 |  | Not reported but the care recipients included parents and grandparents only |  | Not reported | - Work-life balance,  - Flexible working hours and schedule,  - Clear workplace policies on caregiver accommodations,  - Safe and accessible LTC alternatives |
|  | Elliot et al., 2024 | Saskatchewan | Identify and describe existing programs and gaps, create inventories and maps, and explore the service experiences of family caregivers of people living with dementia in these rural areas. | Qualitative - Descriptive (Focus groups, interviews, environmental scan of services, secondary source review (e.g., program brochures), systematic internet search) | 1. Family caregivers of people living with dementia, 2.Healthcare providers/managers | Family caregivers of people living with dementia (n=5), Healthcare providers/managers (n=12) | N= 17 | 61 - 82 | 70 | Not reported |  | Dementia | - Individualized, flexible,  needs-based services,  - Formal and informal supports,  - Dementia-related training and education for service providers,  - Awareness of available services,  - Increasing the number of dementia-related programs with a wide range of schedules and format options. |
|  | Elliott et al., 2022 | Ontario | to develop a Regional Frailty Strategy for Southwestern Ontario by understanding the strengths and gaps in care for older adults living with frailty | Qualitative - Participatory design (Interviews) | Health care providers, including professionals caring for older adults across various roles. Older adults receiving geriatric care and their caregivers with experience managing chronic health conditions. | Health care providers (n=44), including Physicians (n = 10), Nurses (n = 14), Health care administrators (n = 15), and Other health professionals (n = 5). Older adults and caregivers (n=12) | N= 56 | Not reported | Not reported | 65+ |  | A range of health conditions (Chronic), such as Cognitive impairments (e.g., dementia), Falls, Cataracts, Pneumonia, Strokes, Infections, Anxiety, Surgery recovery | - Informational support,  - Patient advocacy while navigating the system,  - Allocation of funding and resources to better support older people,  - Addressing gaps in home care services mainly at policy level. |
|  | Fox et al., 2023 | Ontario | Explore informal rural caregivers' perceived preparedness to detect and respond to the signs of worsening health conditions in patients recently discharged from the hospital and at risk for readmission. | Qualitative - Descriptive (Interviews) | Informal caregivers of a relative discharged from the hospital at high risk for readmission following hospitalization |  | N= 16 | 26 - 67 | 49 | 20 - 87 | 69 | Not reported | - Post-discharge plan for caregivers in rural communities, including: 1) preparedness for post-discharge care to identify and respond to warning signs, and 2) timely access to healthcare/professional resources and support following discharge. |
|  | Fox et al., 2024 | Canada | Illuminate the experiences of patients, living donors, and caregivers on transplant journeys across Canada, to identify the challenges that they face, and to provide recommendations based on their experiences. | Mixed-methods - Parallel design (Survey and focus groups) | Adult patients, living donors, and caregivers | Patients (n=754), donors (n=74), caregivers (n=137) | N= 935 | 18+ |  | 18+ (including 383 patients above 55) |  | Organ transplants | - Financial support,  - Peer support/resources,  - Coordination of care among multiple HCPs, - Mental health support,  - Access to information, care arrangements (e.g., for children), |
|  | Flemons et al., 2022 | Alberta | Examines the experiences and needs of family caregivers (FCGs) for people living with dementia (PLWD) during the coronavirus disease 2019 (COVID-19) pandemic | Qualitative (Online focus groups) | Family caregivers providing care for people living with dementia during the COVID-19 pandemic |  | N= 21 |  | 63 | 71 - 80+ |  | Dementia | Needs during the pandemic:  - Accessible and specific information,  - Centralized information hub for supports and referrals,  - Tailored resources that address both caregivers' and PLWD needs,  - Resources adapted to public health restrictions,  - Physical and mental well-being support (e.g., connecting caregivers with others in similar situations to combat social isolation) |
|  | Garnett et al., 2022 | Ontario | Increase understanding of the factors that influence stroke caregiversâ€™ access and use of formal health and social services, from the perspective of stroke caregivers and healthcare providers. | Qualitative - Interpretive descriptive (In-person or phone interviews) | 1. Stroke caregivers, 2. Health providers who support caregivers and stroke survivors | Stroke caregivers (n=22), Health providers (n=18) | N= 40 | 40 - 89 |  | 30 - 89 |  | Stroke | - Financial support,  - Time for self-care,  - Trust in healthcare providers,  - Information about available services,  - Social support networks. |
|  | Gibson et al., 2025 | Saskatchewan | Explore the experiences and observations of FCs during the COVID-19 prolonged visitor restrictions. | Qualitative (Interviews) | FCs of LTC residents |  | N= 15 | 18+ | 65.4 | Not reported | 88.2 | Not reported | - Recognize FCs as essential to LTC,  - Flexible LTC policies during the pandemic |
|  | Gorenko et al., 2021 | Alberta | Examine the direct influences of caregiving experience, expectations of care, and social support on broader planning outcomes (preparation for aging) | Mixed-method - Sequential explanatory design (Self-report questionnaire followed with interviews) | 1. Caregivers, 2. Caregivers with experience in assisting with an LTC transition, 3. Non-caregivers | Quantitative part: Caregivers (n=74), Caregivers with experience in assisting with an LTC transition (n=92), Non-caregivers (n=180) Qualitative part: Caregivers (n=10), Caregivers with experience in assisting with an LTC transition (n=10) | Quantitative part (N=346), Qualitative part (N=20) | 50+ |  | Not reported |  | Physical illness, mobility issues, cognitive decline or dementia, or a combination. | - Emotional support,  - Informational support,  - Sharing the burden of caregiving,  - Social support in planning for future care needs (e.g., engagement with community organizations) |
|  | Grewal et al., 2024 | Saskatchewan | Understand care partner technology use, attitudes, and the potential role of off-the-shelf technologies (eg, smartphones and smart homes) in supporting caregiving from the perspective of care partners for persons with dementia. | Mixed-methods - Sequential design (survey followed after one year with interviews) | Care partners of people living with dementia |  | N= 67 (survey), N= 10 (interviews) | 18+ |  | Not reported | 82.8 | Dementia | - Facilitating technology use for daily functional and caregiving tasks: supportive teaching or support for setup and troubleshooting, simplicity/ease of use (eg, big screens), failsafe mechanisms in case of internet outages, ability to virtually drop in and check on a loved one, games to facilitate cognitive engagement, ability to set reminders, ability to program phone numbers for voice-activated calls, and any features that would facilitate interaction between care partner and care recipient. |
|  | Guité-Verret et al., 2021 | Quebec | Gain in-depth understanding of family caregivers lived experiences of caregiving and bereavement in the context of the COVID-19 pandemic in Quebec, Canada | Qualitative - Phenomenological approach (Interviews) | Bereaved family caregivers who had lost a loved one during the first waves of the pandemic |  | N= 20 | 21 - 78 | 54.2 | Not reported |  | Mostly with dementia (70%) | - Need for social connections (before, during or after the death of the loved ones),  - Support to cope with pandemic grief, - Clear and compassionate communication |
|  | Hall et al., 2022 | Saskatchewan | Engage caregivers in setting priorities for accessible interventions and support | Qualitative - Descriptive (Focus groups) | Caregivers of older adults |  | N= 33 |  | 79.5 | 55+ |  | Not reported | - Emotional support,  - Informational support (e.g., streamlined website, educational opportunities), - Respite care and self-care activities |
|  | Hall et al., 2024 | Saskatchewan | Explore the lived experiences of family and friend caregivers of older adults and identify their support needs. | Qualitative - descriptive (online survey with open-ended questions) | Family and friend caregivers of older adults |  | N= 354 | 22 - 87 | 61 | 55 - 104 | 83 | The most common medical condition of the care recipient was dementia, followed by heart/kidney/lung conditions, and cancer | - Emotional support,  - Consistency in accessing and receiving healthcare services,  - Targeted supports/interventions to assist caregivers in their caregiving role,  - Governmental systems oriented to aging older adults (financial support, etc.),  - Adequate knowledge on providing care,  - Health literacy |
|  | Hande et al., 2025 | Prince Edward Island and Nova Scotia | Examine how FCs access restrictions in nursing homes that were implemented during the COVID-19 and how they impacted the mutual well-being of and relationships between residents and their caregivers over time. | Qualitative - Longitudinal design (Interviews) | FCs, nursing homes administrators, knowledge users - This study focused on FCs only |  | N= 24 (FCs) | 18+ |  | Not reported |  | Not reported | - Face-to-face communication with LTC residents during the pandemic,  - Supporting social, recreational, and nutritional needs of the residents,  - Advocate for the residents,  - Navigating end-of-life visiting restrictions. |
|  | Holland, 2022 | New Brunswick | Learn more about the lives of people supporting loved ones at home | Qualitative (Interviews, photo elicitation, and observation) | Family caregivers |  | N= 13 | Late 40s - Early 70s |  | Not reported but the care recipients included children |  | Not reported | - Financial and material resources for house improvements/repair |
|  | Isenberg et al., 2021 | Ontario | 1) Explore patients' and caregivers' expectations and subsequent experiences of the hospital-to-home transition while receiving palliative care, and 2) build a substantive grounded theory to enhance the understanding of hospital-to-home transitions from the patient and caregiver perspective. | Qualitative - Longitudinal prospective (Interviews) | 1. Adults receiving inpatient palliative care who were being discharged to home-based palliative care, 2. Family caregivers of adults receiving inpatient palliative care | Adults receiving inpatient palliative care who were being discharged to home-based palliative care (n=25), Family caregivers (of adults receiving inpatient palliative care) (n=14) | N= 39 | 27 - 82 |  | 35 - 96 |  | Cancer, stroke, dementia, pulmonary fibrosis, and other conditions | Needs to be considered in the process of Hospital-to-Home Transition:  - Support with daily activities, such as transportation, setting up the home for care,  - Education about care at home,  -Communication/ coordination of the transition (e.g., additional personnel),  - Community supports,  - Financial resources |
|  | Istanboulian et al., 2024 | Ontario | Explore the impact of care provision on care partners for patients experiencing persistent critical illness. | Qualitative - Descriptive (Interviews) | Adult patients experiencing or who have experienced persistant critical illness admitted to one of the two clinical settings in Toronto, and/or their care partners. | Patients (n=7), care partners (n=9) | N= 16 | 35 - 75 |  | 41 - 80 |  | Not reported | - Self-care activities (e.g., eating healthy, going to the gym),  - Socio-emotional support,  - Financial support,  - Medical knowledge about care recipients' conditions,  - Mental health support,  - Socialization,  - Engagement of care partners in care planning. |
|  | Kokorelias et al., 2022 | Ontario | Develop a conceptual framework of caregiving phases across the Alzheimer's disease and caregiving trajectories and the corresponding caregiver support needs. | Qualitative - Constructivist grounded theory (Phone, in-person or videoconferencing interviews) | Family caregivers (Spousal and adult children) | Spousal caregivers (n=20), Adult children caregivers (n=20) | N= 40 | 45 - 88 |  | Not reported |  | Alzheimer's disease | - Information and education, - Training for caregiving-related activities,  - Navigating healthcare and long-term care, - Respite and self-care,  - Emotional and peer support |
|  | Kuluski et al., 2024 | Canada | Prepare participants to think about safety with a broader lens (beyond just physical harm). | Qualitative - Descriptive (Interviews and focus groups) | Patients, and FCs who were currently using (or had previously used) services in health systems across Canada | Patients (n=14), FCs (n=9), dyads (n=5) | N= 28 | 27 - 73 | 52 | 21 - 65 | 56 | Not reported | - Engaging in meaningful ways with their care teams,  - Emotional support,  - Transparent communication of the healthcare team with patients and caregivers,  - Access to patients' medical records,  - Designating an advocate, such as a point person to communicate with patients and caregivers,  - Educating patients and caregivers on safety. |
|  | Law et al., 2021 | Canada | By using a model for the caregiving role, assess how unpaid caregivers describe their particular roles in caring for a loved one | Qualitative (Interviews) | Unpaid caregivers of people with chronic physical illness |  | N= 39 | 20 - 89 |  | < 20 - 70+ |  | Chronic physical illnesses | - Adequate informational support,  - Adequate training about medical care,  - Recognition for caregivers’ knowledge and expertise,  - Time and support to take care of their own health and social lives,  - Serving as care coordinators for their care recipients with limited authority to make decisions, - Financial and practical assistance,  - Respite care |
|  | Lee, 2020 | Ontario | To examine how end-of-life (EOL) care practices in long-term care (LTC) homes influence caregivers' perceptions of a good resident death. | Cross-sectional (Survey) | Caregivers (family members or friends) of deceased residents who had received end-of-life (EOL) care in LTC homes in Ontario, Canada. |  | N= 78 | 25 to 75+ |  | N/A | N/A | Not reported | - Transparent communication about EOL care,  - Emotional support from LTC staff,  - Involvement in care planning and decisions,  - Attention to spiritual and cultural considerations,  - Assurance of dignified and comfortable care for residents,  - Access to a private and peaceful setting. |
|  | Lane et al., 2022 | Alberta and British Columbia | Examine the prevalence and correlates of anxiety and depressive symptoms among caregivers of assisted living (AL) residents during the initial wave of COVID-19 in two Canadian provinces. | Cross-sectional (Online survey) | Family/friend caregivers | Alberta caregivers (n=546), British Columbia caregivers (n=127) | N= 673 (across 134 participating AL homes) | 18 - 65+ |  | 65+ |  | Dementia, Other mental health conditions such as depression | - Mental health Support,  Financial assistance,  - Improved communication,  - Social and emotional support. |
|  | Legault et al., 2023 | Quebec | Gain insights into the mechanisms and antecedents of presenteeism and absenteeism among employees who are also informal caregivers of seniors | Cross-sectional (Online Questionnaire) | Informal caregivers of seniors |  | N= 915 | 17 - 73 | 43.8 years | Over 65 |  | Not reported | - Workplace flexibility,  - Support for healthcare coordination,  - Financial and job security,  - Better workplace policies,  - Emotional and mental health support |
|  | Lee, 2022 | Quebec | Examine perceptions of caregiving for people living with dementia and help-seeking patterns among prospective Korean caregivers who anticipate becoming primary caregivers of their older relatives with dementia. | Qualitative (Interviews) | Korean caregivers | Korean immigrants (n=6), Canadians of Korean descent (n=3) | N= 9 | 21 - 72 | 33 | Not reported |  | Dementia | - Language and communication assistance,  - Access to resources and formal support, - Access to Korean churches,  - Flexible and culturally adapted support services |
|  | Leslie et al., 2021 | Alberta | Describe family caregivers providing care to older adults approaches to and priorities for achieving care quality and sustainability as they work with formal health and social care systems | Qualitative (Focus groups) | Family caregivers providing care to older adults |  | N= 25 | Not reported |  | Not reported |  | Mostly Alzheimer's disease and dementia | - Improved internal communication with care recipients and external communication with formal system providers,  - Recognition and involvement of caregivers in care planning,  - Access to caregiver-specific training and resources,  - Technology-driven solutions to facilitate information access,  - Caregiver networking and emotional support. |
|  | Leslie et al., 2020 | Alberta | Explore family carers' care-related work goals, and describe how those goals do, or do not, link to technology | Mixed-method - Sequential design (Focus groups followed by online survey). The survey contained an open-ended question. Only responses to this open- ended question were merged with focus group data and included in the qualitative data analysis | Family caregivers providing care to older adults |  | N= 25 (10 focus groups) and N=599 (online survey) | < 65 |  | Over 65 |  | Not reported | - Enhance caregiving capacity about care recipient's condition and systems navigation through technology-based strategies (e.g., help phone lines, interactive applications, context-sensitive search engines),  - Safeguard caregiving capacity and wellbeing through technology-based strategies (e.g., programs connecting caregivers to mentors, counsellors, peer supports) |
|  | Leung et al., 2024 | Ontario | Explain the process taken by Chinese family care partners of older adults in the Greater Toronto Area, Canada, to access health and social services in their communities. | Qualitative - Critical realism approach (Phone or virtual interviews) | Chinese family care partners of older adults |  | N= 28 | 18+ |  | 65+ |  | Not reported | Chinese care partners of older adults' needs:  - Developing self-efficacy by mastering caregiving skills,  - Navigating the healthcare system and community services (e.g., English translators)  - Considering caregivers’ needs, such as flexible workplace, translators, transportation, culturally-appropriate services/LTC homes, etc. |
|  | Li et al., 2023 | Canada | Examine the relationship between geographic distance and caregiver social isolation (CSI), including the interaction between geographic distance and caregiving intensity for CSI. | Cross-sectional (Secondary data analysis of 2012 Canada General Social Survey) | Family caregivers to aging people |  | N= 2,881 | 15+ |  | 65+ |  | Ageing-related frailty, Chronic illnesses, Mobility-related difficulties, Cognitive impairments (e.g., dementia, Alzheimer's disease), Other long-term disabilities | - Reducing social isolation, - Managing caregiving burden and stress,  Support for long-distance caregivers,  - Access to formal support services,  - Emotional/psychological support,  - Financial and employment support |
|  | Li et al., 2023 | Canada | Examine the social participation and social support among older caregivers by comparing 3 types of older caregivers, including spousal caregivers, nonspouse family caregivers, and nonkin (e.g., friends and neighbors) caregivers. | Cross-sectional (Secondary CLSA data analysis) | Older adults who transit into caregiver roles during the two CLSA data collection time points (i.e., noncaregiver at Baseline and caregiver at follow-up 1) | Spousal caregivers (n=1,086), Non-spouse family caregivers (n=1,084), Non-kin caregivers (friends, neighbors, etc.) (n=1,619 ) | N= 3,789 | 65+ |  | Not reported |  | Chronic health conditions or age-related frailty such as Alzheimer's disease, back problems, ulcers, urinary incontinence, etc. | - Programs during the transitional stage, particularly targeting spousal and nonkin older caregivers, to help balance the caregiving role and other out-of-home roles,  - Support for social participation,  - Respite and formal care services,  - Mental health and well-being support |
|  | Li et al., 2024 | Canada | Develop and test the direct and indirect associations between caregiver distress and its many contributing factors and covariates. | Cross-sectional (Secondary data analysis from a national survey) | Unpaid caregivers |  | N= 6,502 | 15+ |  | 15+ |  | Cardiovascular diseases, mental health illness or addiction, cancer, dementia, etc. | - Flexible, holistic and personalized health and social services,  - Interprofessional healthcare team to guide caregivers navigate resources and provide training,  - Transparent and accessible services for caregivers |
|  | Luymes et al., 2021 | Yukon Territory, British Columbia, Alberta, Ontario, Nova Scotia | Identify key factors affecting perceptions of quality palliative care (PC) from the perspective of informal caregivers and decision-makers (e.g., program managers) and to understand how their experiences within the health care system may have influenced their perceptions. | Qualitative (Interviews or focus groups) | 1. Informal caregivers, 2. Decision-makers, including managers, directors and provincial leads, 3. Patients | Informal caregivers (n=9), Decision-makers (n=11), Patients (n=1) | N= 21 | 25 - 81 | 64.6 | Not reported |  | Cancer, kidney failure, heart failure and stroke, etc. | - Patient, caregiver, and family-centred care,  - Timely access to bereavement/PC,  - Anticipatory guidance about caregiver's role, - User-friendly PC system including quality communication with healthcare providers, access to resources, continuity of services, competent staff,  - Support with psychological and spiritual needs of caregivers |
|  | MacLeod et al., 2023 | Ontario | Better understand the health implications of social isolation among community-dwelling older adults and their formal and family caregivers during the pandemic and to make mitigation recommendations | Qualitative - Participatory Action Research (Online or phone interview and policy/procedural document analysis (10 documents)) | Older adults and their family, and/or formal caregivers living within the City or County of Peterborough | Older adults (n=13), Family caregivers (n=9), Formal caregivers (n=9) | N= 31 |  | 68 | 70+ | 86 | The medical conditions lead to limited mobility in older adults, including dementia | - Social support,  - Physical support,  - Mental health support,  - Self-care support,  - Support with technology-based interventions,  - Educational support,  - Financial support,  - Meaningful communication and collaboration between older adults, caregivers, and decision-makers |
|  | Magnaye et al., 2020 | Canada | Better understand family caregivers needs by asking them about their goals in seven life domains: physical health, mental health and well-being, social connections, education, employment, financial well-being, and care-related goals | Cross-sectional (Online survey) | Family caregivers |  | N= 590 | <26 - 85+ |  | Not reported |  | Not reported | - Physical support,  - Mental health and emotional support,  - Financial support,  - Social connection and support networks,  - Employment and career support,  - Education and career-related training |
|  | Marani et al., 2023 | Ontario | Describe the financial risks experienced by unpaid caregivers of persons living with dementia navigating publicly funded homecare in Ontario | Qualitative - Phenomenological approach (Interviews) | Unpaid caregivers of persons living with dementia receiving home care |  | N= 24 | 24 - 81 |  | Not reported |  | Dementia | - Anticipatory information about publicly funded home care available (e.g., PSWs),  - Flexible hours of support from a PSW,  - Consistent access to quality support from a PSW,  - Financial support (PSWs hired privately) |
|  | Marani et al., 2023 | Ontario | Examine and describe the financial risks of unpaid, home-based caregiving throughout the first year of the COVID-19 pandemic | Cross-sectional (Online survey) | Unpaid, home-based caregivers |  | N= 190 | 24 - 93 | 57.8 | Not reported but the care recipients included children |  | Dementia, cancer, arthritis, etc. | - Financial support for care-related expenses,  - Employment flexibility and income security,  - Government and workplace assistance |
|  | McCaughey et al., 2022 | Alberta | Examine how family caregivers for people living with dementia access and evaluate public health messaging in Alberta. | Cross-sectional (Online survey) | Family caregivers of people living with dementia across the care continuum:  at home, in the community, in assisted/supportive living and in long-term care. |  | N= 217 | 50+ |  | 50+ |  | Dementia | - Consistent, tailored, and targeted public health messaging during the pandemic,  - Specific information on how to continue to provide care for a person living with dementia during the pandemic,  - Diverse media sources to communicate messaging to caregivers (e.g., online sources), - Emotional and social support |
|  | McCusker et al., 2020 | Quebec | Describe the unmet needs of caregivers of hospitalized older adults during the transition from hospital back home, and identify subgroups with different needs. | Cross-sectional (Questionnaire) | 1. Family caregivers of older adults who have been hospitalized for an acute medical problem, 2. Care recipients |  | N= 146 patient-caregiver dyads |  | 64 | 65+ |  | Most frequent diagnosis were congestive heart failure, diabetes and pulmonary problems | - Need for patient medical information,  - Need for caregiver role clarity and support,  - Dedicated transitional interventions for this group of caregivers,  - Consideration of the needs of family caregivers in hospital discharge plan |
|  | McKenna et al., 2022 | Canada | Explore multiple sclerosis (MS) caregivers' conceptualizations of resilience, examine MS caregivers experiences of resilience development, and determine which assets and resources influence resilience in this role | Qualitative (Phone or virtual interviews) | Family caregivers of individuals with Multiple Sclerosis |  | N= 24 | 31 - 80 | 56.9 | Not reported but the care recipients included children and siblings |  | Multiple Sclerosis | - Access to reliable information and resources,  - Social connection and peer support,  - Emotional support,  - Respite and self-care opportunities,  - Financial and employment support,  - Caregiving-related training to handle MS-related challenges |
|  | Meng et al., 2025 | British Columbia | Explore the experiences of FCs of people living with dementia | Qualitative - Longitudinal design (Interviews) | FCs of people living with dementia |  | N= 15 | Not reported |  | Not reported |  | Dementia | - Transparency in communication with HCPs,  - Peer support (through professionally led or self-help groups facilitated by other FCs with similar experience),  - Partnering with HCPs for power-sharing (e.g., help in shaping policies),  - Navigating the healthcare system,  - Individualized approach in care,  - Culturally responsive services for caregivers. |
|  | Motta-Ochoa et al., 2021 | Quebec | Explore the intersections of culture and social inclusion/exclusion in a culturally diverse group of persons with dementia, caregivers and staff members | Qualitative - Ethnographic approach (Participant observation and interviews) | 1. People with dementia, 2. Primary caregivers, 3.Staff members of a non-profit organization providing services to people with dementia and their family caregivers | People with dementia (n=31), Primary caregivers (n=9), Staff members (n=9) | N= 49 |  | 66.7 |  | 83.4 | Dementia | - Personal time,  - Social inclusion in the broader community,  - Support networks from family and friends,  - Culturally inclusive and accessible services,  - Health and social organizations with personalized care to promote social inclusion (e.g., accommodate language/cultural needs of clients),  - Guidance in managing dementia progression,  - Recognition of caregivers’ roles and contributions |
|  | Obegu et al., 2025 | Canada | Identify the operational mechanisms of peer support groups and how they influence support for caregivers of people living with sever mental illnesses, by exploring caregivers’ perspectives of peer support groups. | Qualitative (Interviews) | FCs of people living with serious mental illness |  | N= 15 | Not reported |  | Not reported but the care recipients included spouses and siblings |  | Sever mental illnesses | - Non-judgmental messaging/content in peer support groups,  - Support groups for racialized and non-English speaking caregivers,  - Privacy of participants during meetings,  - Accepting the beliefs of the group,  - Having participants of similar age and experience. |
|  | Obegu et al., 2025 | Canada | Understand the perspectives of caregivers about care coordination for people living with serious mental illness, highlighting the current landscape and new directions across Canada | Qualitative - Co-design participatory (Interviews and focus groups) | FCs of people living with serious mental illness, and mental health service providers | FCs (n=18), service providers (n=7) | N= 25 | Not reported |  | Not reported |  | Sever mental illnesses | - Access to and information about mental health services,  - Peer support groups,  - Mental health care coordination,  - Be involved in hospitalization and discharge,  - Designating navigators,  - Trained mental health workers tailored to caregivers,  - Funding for mental health services. |
|  | Peckham et al., 2019 | Ontario | Understand, from the perspectives of caregivers and front line providers, how they access resources, their relationships with (and between) care providers, what resources they need, and how these might inform future policy and programmatic development. | Qualitative - Exploratory (Interviews) | 1. Front-line providers (home and community care providers), 2. Program administrators, 3. Caregivers (both adult child and spousal caregivers) | Front-line providers (n=6), Program administrators (n=9), Caregivers (n=7) | N= 22 |  | 63 |  | Not reported | Not reported | - Have a networks of care (beyond just family members to include friends, neighbors, community organizations, and volunteers),  - Consistency in care to build trusting relationships with providers,  - Interdisciplinary, inter-organizational and inter-sectoral collaboration (integrated system). |
|  | Ravensbergen et al., 2024 | Ontario | Explore double-duty carers' wellbeing during the COVID-19 pandemic, focusing on an understudied factor: their mobility constraints | Qualitative (Virtual interviews) | Double-duty carers (working in a caring role in the healthcare or homecare industry in Ontario and providing unpaid care for a family member or friend) |  | N= 16 | Not reported |  | 18+ (the care recipients included cousins and friends) |  | Not reported | - Supportive work environments to meet the mobility needs of DDCs during the pandemic (e.g., flexible work hours, frequent brief check-ins, access to workplace counselling services, peer-to-peer support groups, and self-care opportunities) |
|  | Reid et al., 2023 | Ontario | Explore the experiences of care partners separated from the person they care for because of institutional visitor restrictions during the COVID-19 pandemic | Qualitative - Descriptive (Online interviews) | Family caregivers of older adults living in an institutional care setting in Ontario, such as a long-term care home, assisted living facility, or retirement community, at some point during the pandemic |  | N= 14 | 50 - 89 |  | Not reported |  | Not reported | - Balanced public health policies,  - Improved and consistent communication with institutions,  - Enhanced emotional and mental health support for caregivers,  - Recognition of caregivers’ contributions to care,  - Training/education to facilitate connection and communication (e.g., assistive technologies for videoconferencing),  - Person- and family-centred approach to care |
|  | Roach et al., 2021 | Alberta | Understand the lived experience of social and physical distancing during the COVID-19 pandemic | Qualitative (Phone interviews) | People living with dementia and family members/care partners who had attended the Cognitive Neurosciences Clinic in Calgary, Alberta |  | N= 21 | Not reported |  |  | 69 | Dementia | - Informal family and community support,  - Formal home care services (e.g., respite care),  - Access to health services, - Assistance with the use of technology in virtual medicine,  - Mental health support. |
|  | Sadavoy et al., 2022 | Canada | Determine the role of organizational variables on employee and work-related outcomes for those employees who provide informal caregiving for older adults, with a special focus on dementia family caregivers | Cross-sectional (Online survey) | Employees who were concurrently providing informal care for an older adult with specific attention to those caring for care recipients with dementia |  | N= 1,839 |  | 49.7 |  | 77.8 | Dementia | - Balancing work and caregiving,  - Family-friendly organizational culture,  - Workplace flexibility,  - Emotional support,  - Financial support |
|  | Sadavoy et al., 2022 | Canada | Determine if employees who are concurrently providing informal caregiving for a person with dementia (DCG) experience greater challenges than employees providing informal caregiving to older adults without dementia (ND CG). | Cross-sectional (Survey) | Informal caregivers of older adults | Informal caregivers of persons with dementia (n=666, with 303 having low dementia demand and 363 having moderate-high dementia demand), Informal caregivers of persons without dementia (n=1,173) | N= 1,839 |  | 49.6 |  | Not reported | Dementia | - Emotional support,  - Access to dementia resource information/support in the workplace,  - Family-friendly work cultures,  - Financial support |
|  | Saragosa et al., 2023 | Canada | Develop a theoretical understanding of the processes people with dementia and their family caregivers engage in during hospital-to-home care transitions | Qualitative - Constructivist grounded theory design (Interviews) | 1. People living with dementia, 2. Family caregivers | People living with dementia (n=4), Family caregivers (n=21) | N= 25 | 21 - 94 | 63.67 | 68 - 91 | 80.75 | Dementia | - Dementia-related information and training,  - Formal and informal support networks,  - Health system navigation and advocacy,  - Financial assistance,  - Respite care,  - Emotional and psychological support,  - Consistent home and community care services |
|  | Savoie et al.,2024 | Quebec | Describe the experience of older persons and caregivers in the transition from driving to ceasing to drive. | Qualitative - Descriptive (Interviews) | 1. Older persons, 2. Informal caregivers | Older persons (n=8), Informal caregivers (n=6) | N= 14 | 56 - 68 |  | 67 - 91 | 78 | Not reported | - Need for information and support regarding the legislation, the process of assessing fitness to drive and alternative means of transportation, such as adapted transportation,  - Emotional support |
|  | Sethi, 2022 | Ontario | Explore the lived experiences of European Transnational Carer Employees (TCEs), defined as immigrants employed in Canada and engaged in caregiving for their aging family members across international borders | Qualitative - Exploratory (Virtual interviews) | European immigrants who are paid workers in Canada and unpaid carers to their family members in their country of origin |  | N= 6 | 18+ |  | Not reported |  | Not reported | - Employment support (e.g., carer-friendly workplace practices),  - Technological support to sustain transnational relationships,  - Social support network in Canada to alleviate caregiving stressors |
|  | Schwarz et al., 2024 | New Brunswick | Examine the experiences of cancer survivors and their caregivers accessing supportive care services as well as gaps and barriers to access | Mixed-methods (survey and open-ended questions in the survey) | Cancer survivors who had received a cancer diagnosis and/or treatment in the past five years, FCs, and care providers | Cancer survivors (n=33), FCs (n=9), and service providers (n=2) | N= 44 | 45 - 54 |  | 55 - 64 |  | Different types of cancer | - Mental health and emotional support,  - Financial assistance,  - Access to cancer-specific information and multidisciplinary services |
|  | Sibalija et al., 2020 | Canada | Investigate the relationships among social support, social participation and depression in caregivers and non-caregivers. | Cross-sectional (Secondary CLSA data analysis) | 1. Caregivers of older adults, 2. Non-caregivers | Caregivers of older adults (n= 3,337), Non-caregivers (n=3,337) | N= 6, 674 | 44 - 87 | 57 | Not reported |  | Not reported | - Social participation (in community-related activities such as recreational sports,  cultural/educationnel activities, etc.),  - Social support |
|  | Silverman, 2021 | Ontario | Examine what dementia-friendly communities could look like from a carer perspective in a Canadian context | Qualitative (Social network maps, mobile interviews, and participant-driven photography) | Family caregivers | Carers of partners/spouses (n=8), Carers of parents (n=4) | N= 12 | 52 - 81 |  | 59 - 90 |  | Dementia | Elements of dementia-friendly communities: 1) accessible and welcoming natural and built environments for shopping, social connections and daily self-care practices,  2) accessible community gathering places for social connections,  3) supportive neighbours and strangers,3) presence of animals, especially dogs, in maintaining social connections. |
|  | Smolej et al., 2023 | Canada | Explore the experiences and support needs of family caregivers who are or who have provided care to individuals who are seeking or have sought medical assistance in dying (MAID). | Qualitative - Descriptive (Phone Interviews with online survey) | Family caregivers supporting individuals living with grievous and irremediable conditions who were seeking or have sought MAID |  | N= 11 | 30 - 68 |  | Not reported but the care recipients included children |  | Dementia, ALS, MS, different types of cancers | - Grief counselling services,  - Peer support groups,  - Self-care activities,  - Advanced care planning for assisting with the administrative aspects of MAID,  - MAID or ‘death coach’ for emotional support,  - Effective communication within the healthcare system about MAID and an end-of-life option |
|  | Stajduhar et al., 2020 | British Columbia | Describe family caregiving in the context of structural vulnerability, to understand who these caregivers are, and the unique challenges, burdens and barriers they face. | Qualitative - Critical ethnography (Observation fieldnotes and interviews) | Family caregivers of structurally vulnerable people who were on palliative care |  | N= 25 | 35 - 74 |  | Not reported |  | Life-limiting conditions such as Arthritis, Cardiovascular disease, Chronic obstructive lung disease (COPD), Cancer, Diabetes, Hepatitis C, HIV/AIDS | Needs-based support programs considering caregiving in the context of 1) poverty and substance use, 2) unstable housing, and 3) complex relational tensions |
|  | Stolee et al., 2021 | Ontario | Present the development of a regional dementia strategy in Southwestern Ontario, Canada | Qualitative (Environmental scan, interviews, priority-setting survey) | 1. Persons with dementia, 2. Care partners of persons with dementia, 3. Healthcare administrators and policymakers | For interviews: Persons with dementia (n=6), Care partners of persons with dementia (n = 20), Healthcare administrators and policymakers (n = 33) For surveys: Persons with dementia/care partners (n=12), Healthcare providers/administrators (n=52) | N= 59 (Interviews), N= 64 (Surveys) | 56 - 80 |  | 59 - 94 |  | Dementia | - Support with care coordination and system navigation,  - Person-centred care,  - Flexibility in services,  - Training/education for providers, patients, care partners and the public |
|  | Sun et al., 2021 | Ontario | Examine the perspectives of homecare clients and their informal caregivers about the role of therapeutic self-care in supporting safety in homecare | Qualitative - Descriptive (Interviews) | 1. Older homecare clients, 2. Informal caregivers (clients' caregivers) | Older homecare clients (n=15), Informal caregivers (n=15) | N= 30 | Not reported | 54 | 65+ | 72 | Not reported | - Physical support,  - Emotional support,  - Financial support,  -Social support,  - Support with environmental/safety issues,  - Technological support, |
|  | Tam et al., 2021 | British Columbia | By using a patient-centred care, explore the experiences and needs of people living with dementia and their care partners during the COVID-19 pandemic as part of an ongoing evaluation of dementia support services | Cross-sectional (Online survey) | Individuals who have lived experience with dementia or care partner of people with dementia | Care partners (n=395), Individuals with lived experiences of dementia (n=22) | N= 417 | 20 - 80+ |  | 60 - 80+ |  | Dementia | - Information and resource support (e.g., high-quality, evidence-based information about care resources and the pandemic),  - Mental health and well-being support (e.g., social support systems, social connection),  - Need to understand the barriers to using technology as a source of social connection,  - Need to address and understand the COVID-19 related concerns (e.g., visitation rules of LTC homes) |
|  | Tseung et al., 2020 | Ontario | Explore factors affecting implementation of caregiver support programmes in healthcare institutions in a regional stroke system | Qualitative - Descriptive (Interviews and focus groups) | Ontario Stroke System stakeholders, including medical directors, executives, programme directors, education coordinators, rehabilitation and community and long-term care specialists, primary care leaders and healthcare professionals | Regional/district programme directors (n=19), Community and long-term care specialists (n=7), Regional education coordinators (n=7), Regional rehabilitation specialists (n=11), Regional medical directors (n=2), Health region executives (n=4), Health region primary care leads (n=4), Healthcare professionals (n=19) | N= 72 (43 participated in focus groups and 29 participated in individual interviews) | N/A |  | N/A |  | Stroke | - Availability of evidence to support the outcomes of programs intended for caregivers,  - Personnel requirements,  - Supportive structure including billing, workflow and resources |
|  | Wang et al., 2022 | Ontario | Explore the experiences of family caregivers of persons living with dementia on using apps in their caregiving roles. | Qualitative - Descriptive (Interviews and photo elicitation) | Family caregivers of persons living with dementia (Adult child family caregivers only) |  | N= 5 | 18 - 35 |  | 51 - 90 |  | Dementia | - Apps adapted to the needs of the dyad,  - Features of effective: 1) simple and easy to navigate, 2) interactive to share relevant information with other family members, 3) accessible from various mobile devices, 4) have multiple language options, 5) multifunctional to address the needs of caregivers |
|  | Webber et al., 2024 | Ontario | Explore and comprehend the lived experiences and realities of caregivers based in Northern Ontario | Qualitative - Descriptive design (focus groups) | FCs |  | N= 36 | 25 - 83 | 54 | Not reported |  | Not reported | - Being recognized as an essential part of the healthcare team,  - Financial support,  - Socialization,  - Mental/emotional support,  Technological/digital support for virtual appointments during the pandemic,  Respite care,  - Personalized support,  - Access to relevant resources/services for caregivers (e.g., local community organizations, government resources, etc.),  - Culturally sensitive care,  - Targeted training and support for caregivers,  - Support groups and mentorship opportunities,  - Training for HCPs about the role of caregivers |
|  | Weeks et al., 2021 | Ontario and Nove Scotia | Identify the health service experiences and preferences of frail home care clients and their family and friend caregivers during the COVID-19 pandemic | Qualitative (Phone interviews) | 1. Home care clients, 2. Caregivers (Clients' caregivers) | Home care clients (n=10), Caregivers (n=19) | N= 29 | Not reported |  | Not reported |  | Not reported | - Technology-based resources (e.g., passive RMT),  - Enhanced regulations to consider LTC placements,  - Consistent home care services/providers |
|  | Williams et al., 2022 | Ontario | Identify and develop standardized quality indicators (QIs) for community-based palliative care in Canada to improve care quality, address gaps, and optimize outcomes for individuals with serious illnesses and their caregivers. | Qualitative (One-day workshop with three brainstorming sessions) | Stakeholders in palliative care, such as clinical leaders, researchers, frontline staff, health and information system administrators, and decision-makers |  | N=30 | N/A | N/A | N/A | N/A | Not reported | - Enhanced education and training to equip caregivers with the skills,  - Access to strong support systems, including respite services and emotional support networks,  - Clear communication,  - Inclusion in care planning |
|  | Wilson et al., 2025 | Alberta | Identify the needs and support services for FCs of older community-based family members | Qualitative (Interviews) | FCs of older community-based family members |  | N= 150 | 22 - 86 | 66 | 65+ |  | Not reported | - Informational,  - Emotional,  - Help with care recipient's instrumental activities of daily living,  - Help with care recipient activities of daily living,  - Respite/self-care encouragement,  - Transportation assistance,  - Financial help with care at home costs,  - Anticipatory grief of the family caregiver,  - Support after their caregiving ends |
|  | Xiong et al., 2020 | Canada | Examine (1) the knowledge of technology, (2) perceived usefulness of technology, (3) feature preferences when installing and using technology and (4) sex and gender influences on technology needs and preferences among family caregivers of persons with dementia (PWD) across North America | Cross-sectional (Questionnaire completed electronically, by paper, in-person, or over the phone) | Family caregivers of people living with dementia living in North America |  | N= 381 | 20 - 94 | 62.6 |  | 78.6 | Dementia | - Accessibility, knowledge, and awareness of technology in caregiving,  - Considering the needs and preferences of caregivers in developing technology to support them (e.g., cost, manual input) |
|  | Yagelniski et al., 2020 | Saskatchewan | Explore experiences and perceptions of lung transplant caregivers identified from a satellite clinic to inform the development of educational resources. | Qualitative - Phenomenological approach (Phone interviews) | Caregivers of patients who were either in the assessment process or had previously undergone lung transplantation through the Saskatchewan Lung Transplant Program (SLTP) in Saskatoon |  | N= 12 | 50 - 74 | 64.6 | Not reported |  | Lung transplantation | - Social support,  - Emotional support,  - Physical and daily life support,  - Support networks,  - Educational support |
|  | Yakerson, 2022 | Ontario | Examine the lived experiences of informal family caregivers who seek in-home publicly funded care for their relatives. | Qualitative - Phenomenological approach (Interviews) | Informal family caregivers receiving home care services funded publicly by the Local Health Integration Networks (LHINs) within the Greater Toronto Atea |  | N= 12 | Not reported |  | Not reported |  | Not reported | - Support in finding out about and accessing home care services,  - Support with navigating the system when changing region/LHINs, - Respite needs (e.g., sufficient hours of care and consistency in care),  - Services based on clients' needs (e.g. language needs),  - Psychological support,  - Financial support |
|  | Yang et al., 2021 | Quebec | Examine to what extent the needs differ between Canadian- and foreign-born patients and caregivers and investigate their experiences with the illness in primary care case management. | Mixed-method - Sequential explanatory design (Cross-sectional survey followed by qualitative interviews) | 1. Canadian-born and foreign-born Patients, 2. Canadian-born and foreign-born informal caregivers |  | N= 30 (15 pairs of patients and caregivers) | 41 - 90 | 72.3 | 61 - 91 | 76.5 | Dementia | Needs of foreign-born caregivers:  - Respite care (e.g., patient care in a day centre),  - Designating a case manager |

**Table S3—**Summary of Included Studies (systematic review, Canada, 2020–2025).
