# Supplementary material for: Unveiling the Hidden Challenges: A Systematic Review of Self-Identified Caregiver Support Needs for Older Adults in Canada
Source: Public Health Rev. 2026 Feb 26;47:1609117. doi: 10.3389/phrs.2026.1609117 (PMC12979237; doi:10.3389/phrs.2026.1609117)
Supplement: Supplementary file 4 [file Table4.docx]

**Supplementary Table S4—Overview of themes and sub-themes**

| Major themes | Sub-themes | Key terms/phrases |
| --- | --- | --- |
| Equipping Caregivers to Navigate the System through Information Access and Education | Clear, Contextualized, and Centralized Information  (1–29) | - Information on access to health and social services  - Context-specific information  - Evidence-based information about the pandemic |
|  | Education and Skill Development (3,6,10,12,15,17,20–22,30–50) | - Clear home care instructions following hospital-to-home transition  - Caregivers’ training to enhance health literacy  - Self-efficacy by mastering caregiving skills  - Consistent and proactive information on care recipient’s health condition  - Access to educational resources during the pandemic  - Preparedness for post-discharge care  - Information about stages of dementia |
| Addressing Caregivers’ Evolving Needs Through Accessible and Inclusive Technology  (19,23,25,35,38,39,41,50–57) |  | - Improving digital literacy  - Inclusive digital innovations  - Support with technology-based interventions during the pandemic  - Frequent and regular videoconferencing meetings/sharing video and photos of LTC residents with care partners |
| Empowering caregivers in a coordinated and integrated care system to meet individual needs | Integrated, Transparent, and Competent Communication Across Care Settings  (9,24,27,30,34–36,39–43,48,56,58–66) | - Interprofessional and inter-organizational collaboration  - Clear and meaningful communication, especially during the pandemic  - Competent staff  - Proactive communication with care partners  - Transparency in communication among LTC staff |
|  | Caregivers as Essential Partners in Collaborative Care  (5,8,10,27,28,32,39,41,42,44,47,48,50,54,56,62–64,66–69) | - Caregivers as part of the care team  - Recognition for caregivers’ knowledge and expertise  - Caregivers as 'essentials' rather than 'visitors' during the pandemic  - Partnering with HCPs for power-sharing (e.g., help in shaping policies) |
|  | Supportive Roles to Inform and Empower Family Caregivers (2,24,28,39,48,60,65,70) | - Allocate supportive roles such as case managers and system navigators |
|  | Responsive and Adaptive Care Structure  (1–3,6–8,20,21,24,27,28,30,33,36,38–40,42,44,45,50,51,54–56,64–66,70–75) | - Evidence-based programme implementation  - Training/education to HCPs  - Address human resources challenges, such as a lack of staff, stable care teams, and qualified professionals  - Consistency in accessing and using services, including palliative care and home care  - Sufficient/flexible hours of home care services  - Individualized, flexible, needs-based approaches  - Culturally and linguistically adapted support services  - Flexible services for caregivers in different contexts, such as poverty, substance use, and unstable housing. |
|  | Flexible and Needs-based Visitation Policies in Times of Crisis (21–23,39,54,56,63,67,69,76) | - Revision of pandemic visitation restrictions  - Consistent/clear visitation and public health policies  - Be able to support the social, recreational, and nutritional needs of the residents during the pandemic |
| Supporting the Emotional, Social, and Practical Well-being of Family Caregivers Across the Care Journey | Emotional and Mental Health Support  (1,4,5,7,12–19,22,23,26,29–36,38,39,41,43,47,49,50,56,58,61,62,64,68,70,71,74,77,78) | - Emotional/mental health support, including during the pandemic  - Need for acknowledgement of caregivers' struggles  - Empathy in healthcare providers' interactions  - Support to cope with pandemic grief |
|  | Social Connections and Community-Based Support Networks  (4,9,13,15,18,19,21–24,26,30,31,35,38,39,41,42,44,47,50,52,58,61,66,68,70–72,77,79) | - Peer support  - Community support (e.g., local churches, social participation in community-based activities)  *-* Social health support during the pandemic  - Informal support from family and friends  - Connecting caregivers with others in similar situations (e.g., similar age and experience) |
|  | Practical Relief Through Accessible Services and Respite Options  (1,5,7–10,12,15,17,19,20,22,24,29–32,35,38–40,44,47,50,60,68,70,71,77,78) | - Home care services  - Patient care in a day centre/adult day programs  - Personal time for caregivers  - Respite as a critical priority  - Support with physical care and basic activities of daily living, such as bathing, toileting, feeding, and mobility support  - Support with Instrumental Activities of Daily Living (IADLs), such as managing transportation, shopping, home maintenance, and managing medication. |
| Enhancing Financial and Workplace Supports for Family Caregivers Across Diverse Contexts | Addressing the Financial and Material Burdens of Caregiving  (1,2,4,5,8–10,13,15,16,22,26,29,30,33,35,38,39,47,49,61,68,74,77,78,80,81) | - Support to address financial costs and material strain, such as hiring PSWs privately, purchasing medical equipment, and house improvements/repairs  - Government financial support  - Financial resources during the pandemic  - Financial and material resources for rural caregivers |
|  | Caregiver-Friendly Workplaces and Flexible Employment Policies  (5,15,16,20,52,74,77,78,81–83) | - Flexible working hours  - Access to support resources in the workplace  - Employment support for caregivers with special circumstances, such as transnational caregivers (employed caregivers caring for loved ones across international borders) and double-duty carers (individuals who provide paid care as part of their healthcare employment and unpaid care to a relative or friend outside of work.  - Balancing work and family |

**Table S4 |** Overview of themes and sub-themes

**References**

1. Yakerson A. Informal family caregiver experiences with publicly funded home care in Ontario. Home Health Care Serv Q. 2022;41(1):65–75.

2. Marani H, Shaw J, Marchildon GP. Challenges navigating publicly funded home care in Ontario, Canada: Perspectives from unpaid caregivers of persons living with dementia. Dementia. 2023 Oct 1;22(7):1626–45.

3. McCusker J, Yaffe M, Lambert SD, Cole M, de Raad M, Belzile E, et al. Unmet needs of family caregivers of hospitalized older adults preparing for discharge home. Chronic Illn. 2020 Jun 1;16(2):131–45.

4. Chappell NL, Penning M, Kadlec H, Browning SD. Care-giver wellbeing: exploring gender, relationship-to-care-recipient and care-giving demands in the Canadian Longitudinal Study on Aging. Ageing Soc. 2023;43(11):2517–53.

5. Cruz E, Paré MA, Stan C, Voth J, Ward L, Taboun M. Caring for the caregiver: An exploration of the experiences of caregivers of adults with mental illness. SSM - Qualitative Research in Health. 2024;5:100406.

6. Li W, Manuel DG, Isenberg SR, Tanuseputro P. Using Exploratory Structural Equation Modeling to Examine Caregiver Distress and Its Contributors. J Am Med Dir Assoc. 2024 May 1;25(5):817-825.e5.

7. Flemons K, McGhan G, McCaughey D. Family Caregiving for People Living With Dementia During COVID-19: A Thematic Analysis. J Fam Nurs. 2022 Aug 1;28(3):219–30.

8. Cooper EJ, Sanguins J, Menec V, Chartrand AF, Carter S, Driedger SM. Culturally responsive supports for metis elders and metis family caregivers. Canadian Journal on Aging. 2020 Jun 1;39(2):206–19.

9. Garnett A, Ploeg J, Markle-Reid M, Strachan PH. Factors impacting the access and use of formal health and social services by caregivers of stroke survivors: an interpretive description study. BMC Health Serv Res. 2022 Dec 1;22(1).

10. Law S, Ormel I, Babinski S, Kuluski K, Quesnel-Vallée A. “Caregiving is like on the job training but nobody has the manual”: Canadian caregivers’ perceptions of their roles within the healthcare system. BMC Geriatr. 2021 Dec 1;21(1).

11. Barber B, Weeks L, Steeves-Dorey L, McVeigh W, Stevens S, Moody E, et al. Hospital to Home: Supporting the Transition From Hospital to Home for Older Adults. Canadian Journal of Nursing Research. 2022 Dec 1;54(4):483–96.

12. Hall S, Holtslander L. Assessing the Need for Caregiver Support in Saskatchewan, Canada: Gathering Perspectives and Setting Priorities. Canadian Geriatrics Journal. 2022 Sep 1;25(3):233–9.

13. Li L, Wister A. Geographic distance and social isolation among family care-givers providing care to older adults in Canada. Ageing Soc. 2023 Feb 10;43(2):298–323.

14. Savoie C, Voyer P, Lavallière M, Bouchard S. Transition from driving to driving-cessation: experience of older persons and caregivers: a descriptive qualitative design. BMC Geriatr. 2024 Dec 1;24(1).

15. McKenna O, Fakolade A, Cardwell K, Pilutti LA. A continuum of languishing to flourishing: exploring experiences of psychological resilience in multiple sclerosis family caregivers. Int J Qual Stud Health Well-being. 2022;17(1).

16. Beauchamp Legault MÈ, Chênevert D, Maisonneuve F, Mansour S. How do Informal Caregivers of Seniors’ Tasks Lead to Presenteeism and Absenteeism Behaviors? A Canadian Quantitative Study. Int J Environ Res Public Health. 2023 Apr 1;20(7).

17. Kokorelias KM, Gignac MAM, Naglie G, Rittenberg N, MacKenzie J, D’Souza S, et al. A grounded theory study to identify caregiving phases and support needs across the Alzheimer’s disease trajectory. Disabil Rehabil. 2022;44(7):1050–9.

18. Gorenko JA, Konnert C, Speirs C. Does Caregiving Influence Planning for Future Aging?: A Mixed Methods Study Among Caregivers in Canada. Res Aging. 2021;43(5–6):203–13.

19. Roach P, Zwiers A, Cox E, Fischer K, Charlton A, Josephson CB, et al. Understanding the impact of the COVID-19 pandemic on well-being and virtual care for people living with dementia and care partners living in the community. Dementia. 2021 Aug 1;20(6):2007–23.

20. Leung DYL, Lee CT, Chu SYJ, Ng F, Wen P, Fan J, et al. Chinese family care partners of older adults in Canada have grit: A qualitative study. J Adv Nurs. 2024 Mar 1;80(3):1018–29.

21. Elliot V, Kosteniuk J, O’Connell ME, Cameron C, Morgan D. Services for older adults in rural primary care memory clinic communities and surrounding areas: a qualitative descriptive study. BMC Health Serv Res. 2024 Dec 1;24(1).

22. Saragosa M, Kuluski K, Okrainec K, Jeffs L. “Seeing the day-to-day situation”: A grounded theory of how persons living with dementia and their family caregivers experience the hospital to home transition and beyond. J Aging Stud. 2023 Jun 1;65.

23. Tam MT, Dosso JA, Robillard JM. The Impact of a Global Pandemic on People Living with Dementia and Their Care Partners: Analysis of 417 Lived Experience Reports. J Alzheimers Dis. 2021;80(2):865–75.

24. Elliott J, Koch M, McDermott M, Sacco V, Stolee P. Developing a Regional Strategy for Older Adults Living With Frailty: Recommendations From Patients, Family Caregivers and Health Care Providers. Int J Integr Care. 2022;22(3):13.

25. Leslie M, Gray RP, Eales J, Fast J, Magnaye A, Khayatzadeh-Mahani A. The care capacity goals of family carers and the role of technology in achieving them. BMC Geriatr. 2020 Feb 27;20(1).

26. Fox DE, Hall M, Thibodeau C, Coldwell K, Lauder L, Dewell SL, et al. The experiences of patients, caregivers and donors on transplant journeys in Canada: A convergent parallel mixed methods study. J Eval Clin Pract. 2024 Mar;30(2):268–80.

27. Meng C, Lachapelle S, Adekoya A, Kervin L, Seetharaman K, Basu Khan K, et al. Using a Trauma-Informed Care Approach to Understand Family Caregivers’ Experiences of Accessing Formal Supports in Dementia Care. J Fam Nurs. 2025 Feb;31(1):3–15.

28. Obegu P, Nicholls K, Alberti M. Care coordination for people living with serious mental illness: understanding the caregiver’s perspective. Frontiers in health services. 2025;4:1473235.

29. Wilson DM, Heron J, Banamwana G. Identifying Needs and Support Services for Family Caregivers of Older Community-Based Family Members: Mixed-Method Research Findings. Journal of Applied Gerontology. 2025;

30. Isenberg SR, Killackey T, Saunders S, Scott M, Ernecoff NC, Bush SH, et al. “Going Home [Is] Just a Feel-Good Idea With No Structure”: A Qualitative Exploration of Patient and Family Caregiver Needs When Transitioning From Hospital to Home in Palliative Care. J Pain Symptom Manage. 2021 Sep 1;62(3):e9–19.

31. Yagelniski A, Rosaasen N, Cardinal L, Fenton ME, Tam J, Mansell H. A Qualitative Study to Explore the Needs of Lung Transplant Caregivers. Progress in Transplantation. 2020 Sep 1;30(3):243–8.

32. Williams N, Boumans N, Luymes N, White NE, Lemonde M, Guthrie DM. What should be measured to assess the quality of community-based palliative care? Results from a collaborative expert workshop. Palliat Support Care. 2022;20(2):226–32.

33. Hall S, Rohatinsky N, Holtslander L, Peacock S. Challenges Reported by Family and Friend Caregivers to Older Adults in the Saskatchewan Caregiver Experience Study. Home Health Care Manag Pract. 2025;37(1):3–13.

34. Dale CM, Carbone S, Istanboulian L, Fraser I, Cameron JI, Herridge MS, et al. Support needs and health-related quality of life of family caregivers of patients requiring prolonged mechanical ventilation and admission to a specialised weaning centre: A qualitative longitudinal interview study. Intensive Crit Care Nurs. 2020;58:102808.

35. Macleod A, Levesque J, Ward-Griffin C. Social Isolation of Older Adults, Family, and Formal Caregivers during the COVID-19 Pandemic: Stories and Solutions Through Participatory Action Research. Canadian Journal on Aging. 2023;

36. Luymes N, Williams N, Garrison L, Goodridge D, Silveira M, Guthrie DM. “The system is well intentioned, but complicated and fallible” interviews with caregivers and decision makers about palliative care in Canada. BMC Palliat Care. 2021 Dec 1;20(1).

37. Fox MT, Butler JI. Rural caregivers’ preparedness for detecting and responding to the signs of worsening health conditions in recently hospitalised patients at risk for readmission: A qualitative descriptive study. BMJ Open. 2023 Dec 28;13(12).

38. Sun W, Ashtarieh B, Zou P. The safety challenges of therapeutic self-care and informal caregiving in home care: A qualitative descriptive study. Geriatr Nurs (Minneap). 2021 Mar 1;42(2):491–501.

39. Bourbonnais A, Lachance G, Baumbusch J, Hsu A, Daneau S, Macaulay S. At the Epicentre of the COVID-19 Pandemic in Canada: Experiences and Recommendations of Family Care Partners of an Older Person Living in a Long-Term Care Home. Canadian Journal on Aging. 2024 Jun 1;43(2):244–56.

40. Bélanger-Dibblee M, Pham Thi-Desmarteau S, Jacques MC, Tremblay H, Roy-Desruisseaux J. The Experiences, Needs, and Solutions of Caregivers of Patients With Behavioral and Psychological Symptoms of Dementia Living in Residential and Long-Term Care Centers. Qual Health Res. 2023 Aug 1;33(10):871–83.

41. Leslie M, Gray RP, Khayatzadeh-Mahani A. What is “care quality” and can it be improved by information and communication technology? A typology of family caregivers’ perspectives. Scand J Caring Sci. 2021;35(1):220–32.

42. Ashbourne J, Boscart V, Meyer S, Tong CE, Stolee P. Health care transitions for persons living with dementia and their caregivers. BMC Geriatr. 2021 Dec 1;21(1).

43. McCaughey D, McGhan G, Flemons K, Hindmarch W, Brundrit K. Public Health Messaging during the COVID-19 Pandemic and Its Impact on Family Caregivers’ COVID-19 Knowledge. Healthcare policy. 2022;18(1):75–89.

44. Motta-Ochoa R, Bresba P, Da Silva Castanheira J, Lai Kwan C, Shaffer S, Julien O, et al. “When I hear my language, I travel back in time and I feel at home”: Intersections of culture with social inclusion and exclusion of persons with dementia and their caregivers. Transcult Psychiatry. 2021 Dec 1;58(6):828–43.

45. Stolee P, Ashbourne J, Elliott J, Main S, Holland N, Edick C, et al. Whole Person, Whole Journey: Developing a Person-Centred Regional Dementia Strategy. Canadian Journal on Aging. 2021 Sep 1;40(3):436–50.

46. Abuzuluf H, Giannopoulos E, Bradbury P, Doherty M, Donahoe L, Czarnecka-Kujawa K, et al. Informational Needs of Lung Cancer Patients and Caregivers. J Cancer Educ. 2025 Jan 6;

47. Istanboulian L, Gilding AJ, Hamilton L, Master T, Bingler S, Soldatic K, et al. Reported impact and protective factors of the care partner role during persistent critical illness: a content analysis. BMC Nurs. 2024 Sep 6;23(1):625.

48. Kuluski K, Asselbergs M, Baker R, Burns KKK, Bruno F, Saragosa M, et al. “Safety is about partnership”: Safety through the lens of patients and caregivers. Health Expect. 2024 Feb;27(1):e13939.

49. Schwarz C, Luke A, Besner J, MacNeill L, Ashfield LR, Easley J, et al. Exploring the experiences of cancer survivors and their caregivers accessing supportive care services in New Brunswick, Canada. Can Oncol Nurs J. 2024;34(4):523–38.

50. Webber J, Mulroney E, Tatasciore M, Smith B, Duggan PJ, Ferron L, et al. Post-Pandemic Needs of Unpaid Family and Friend Caregivers to Effectively Continue Caregiving Duties in one Northern Ontario Health Authority. Patient Exp J. 2024;11(3):106–16.

51. Xiong C, Ye B, Mihailidis A, Cameron JI, Astell A, Nalder E, et al. Sex and gender differences in technology needs and preferences among informal caregivers of persons with dementia. BMC Geriatr. 2020 May 18;20(1).

52. Sethi B. Negotiating culture, geographical distance, and employment: The lived experiences of European transnational carer employees. Wellbeing, Space and Society. 2022 Jan 1;3.

53. Wang AH, Newman K, Martin LS, Lapum J. Beyond instrumental support: Mobile application use by family caregivers of persons living with dementia. Dementia. 2022 Jul 1;21(5):1488–510.

54. Boamah SA, Weldrick R, Yous ML, Gao H, Garnett A, Bello-Haas VD, et al. “Picturing a Way Forward”: Strategies to Manage the Effects of COVID-19-Related Isolation on Long-Term Care Residents and Their Informal Caregivers. Gerontologist. 2024 Jan 1;64(1).

55. Weeks LE, Nesto S, Hiebert B, Warner G, Luciano W, Ledoux K, et al. Health service experiences and preferences of frail home care clients and their family and friend caregivers during the COVID-19 pandemic. BMC Res Notes. 2021 Dec 1;14(1).

56. Reid JC, Carbone S, Shaw JF, Gallibois M, Hawkins SA. “My Biggest Fear Is She’ll Die Alone”: Care Partner Perspectives of Institutional COVID-19 Visitor Restrictions in Ontario, Canada. Can J Aging. 2023;42(4):710–8.

57. Grewal KS, Gowda-Sookochoff R, Peacock S, Cammer A, McWilliams LA, Spiteri RJ, et al. Perspectives on Technology Use in the Context of Caregiving for Persons With Dementia: Qualitative Interview Study. JMIR Form Res. 2024 Dec 13;8:e63041.

58. Guité-Verret A, Vachon M, Ummel D, Lessard E, Francoeur-Carron C. Expressing grief through metaphors: family caregivers’ experience of care and grief during the Covid-19 pandemic. Int J Qual Stud Health Well-being. 2021;16(1).

59. Sibalija J, Savundranayagam MY, Orange JB, Kloseck M. Social support, social participation, & depression among caregivers and non-caregivers in Canada: a population health perspective. Aging Ment Health. 2020;24(5):765–73.

60. Yang XQ, Vedel I, Khanassov V. The cultural diversity of dementia patients and caregivers in primary care case management: A pilot mixed methods study. Canadian Geriatrics Journal. 2021 Sep 2;24(3):184–94.

61. Lane NE, Hoben M, Amuah JE, Hogan DB, Baumbusch J, Gruneir A, et al. Prevalence and correlates of anxiety and depression in caregivers to assisted living residents during COVID-19: a cross-sectional study. BMC Geriatr. 2022;22(1):662.

62. Conklin J, Dehcheshmeh MM, Archibald D, Elliott J, Hsu A, Kothari A, et al. From Compliance to Care: Qualitative Findings from a Survey of Essential Caregivers in Ontario Long-Term Care Homes. Can J Aging. 2024;43(4):538–47.

63. Boamah SA, Yous M, Gao H, Weldrick R, Dal Bello‐Haas V, Durepos P. Insight into the experiences of caregivers of older adults in long‐term care homes: A photovoice study. J Adv Nurs. 2024;80(3):1084–96.

64. Lee E, Sussman T, Kaasalainen S, Durepos P, McCleary L, Wickson-Griffiths A, et al. The relationship between caregivers’ perceptions of end-of-life care in long-term care and a good resident death. Palliat Support Care. 2020;18(6):683–90.

65. Tseung V, Jaglal S, Salbach NM, Cameron JI. A Qualitative study assessing organisational readiness to implement caregiver support programmes in Ontario, Canada. BMJ Open. 2020;10(5):e035559.

66. Peckham A, Williams P, Denton M, Berta W, Kuluski K. “It’s More than Just Needing money”: The Value of Supporting Networks of Care. J Aging Soc Policy. 2019;

67. Chu CH, Yee A V., Stamatopoulos V. “It’s the worst thing I’ve ever been put through in my life”: the trauma experienced by essential family caregivers of loved ones in long-term care during the COVID-19 pandemic in Canada. Int J Qual Stud Health Well-being. 2022;17(1).

68. Anderson S, Parmar J, Dobbs B, Tian PGJ. A tale of two solitudes: Loneliness and anxiety of family caregivers caring in community homes and congregate care. Int J Environ Res Public Health. 2021 Oct 1;18(19).

69. Gibson K, Alford H, Ward H, Hunter P V. “Families are a resource, not the enemy”: Canadian family caregivers’ experiences of COVID-19 pandemic visitor restrictions. J Aging Stud. 2025 Mar;72:101311.

70. Smolej E, Malozewski M, McKendry S, Diab K, Daubert C, Farnum A, et al. A qualitative study exploring family caregivers’ support needs in the context of medical assistance in dying. Palliat Support Care. 2023;21(2):254–60.

71. Li L, Wister A V., Lee Y, Mitchell B. Transition Into the Caregiver Role Among Older Adults: A Study of Social Participation and Social Support Based on the Canadian Longitudinal Study on Aging. Journals of Gerontology - Series B Psychological Sciences and Social Sciences. 2023 Aug 1;78(8):1423–34.

72. Lee E. Perceptions of caregiving for people living with dementia and help-seeking patterns among prospective Korean caregivers in Canada. Health Soc Care Community. 2022 Nov 1;30(6):e4885–93.

73. Stajduhar KI, Giesbrecht M, Mollison A, Dosani N, McNeil R. Caregiving at the margins: An ethnographic exploration of family caregivers experiences providing care for structurally vulnerable populations at the end-of-life. Palliat Med. 2020 Jul 1;34(7):946–53.

74. Sadavoy J, Sajedinejad S, Duxbury L, Chiu M. The impact on employees of providing informal caregiving for someone with dementia. Aging Ment Health. 2022;26(5):1035–43.

75. Silverman M. Dementia-Friendly Neighbourhoods in Canada: A Carer Perspective. Vol. 40, Canadian Journal on Aging. Cambridge University Press; 2021. p. 451–62.

76. Hande MJ, Weeks LE, Chamberlain SA, Hubley E, Burke R, Warner G, et al. Caregivers’ Experiences of Nursing Home Restrictions During the COVID-19 Pandemic. Can J Aging. 2025 Jan 6;1–10.

77. Magnaye A, Fast J, Eales J, Stolow M, Leslie M. Caregivers’ failure to thrive: A case for health and continuing care systems transformation. Healthc Manage Forum. 2020 Sep 1;33(5):214–9.

78. Sadavoy J, Sajedinejad S, Duxbury L, Chiu M. A Canadian national survey of informal employed caregivers of older adults with and without dementia: Work and employee outcomes. Int J Soc Psychiatry. 2022;68(1):183–95.

79. Obegu P, Nicholls K, Alberti M. Operational Mechanisms of Peer Support Groups and Support for Caregivers of People Living with Serious Mental Illness. Community Ment Health J. 2025 Jan;61(1):59–65.

80. Holland MR. More than chores: The invisible health work of family caregivers in rural New Brunswick, Canada. Health Place. 2022 Jan 1;73.

81. Marani H, Allin S, McKay S, Marchildon GP. The Financial Risks of Unpaid Caregiving During the COVID-19 Pandemic: Results From a Self-reported Survey in a Canadian Jurisdiction. Health Serv Insights. 2023 Jan 1;16.

82. Ding RY, Williams AM. Places of paid work and unpaid work: Caregiving and work-from-home during COVID-19. Canadian Geographer. 2022 Mar 1;66(1):156–71.

83. Ravensbergen L, Mehta S, Sethi B, Ward-Griffin C, Williams A. Double-Duty Carers’ Health and Wellbeing during COVID-19: Exploring the Role of Mobility of the Care Economy in Southern Ontario, Canada. Int J Environ Res Public Health. 2024 Jun 1;21(6).
